# A Systematic Review of Mortality from Untreated Scrub Typhus (*Orientia tsutsugamushi*)

**DOI:** 10.1371/journal.pntd.0003971

**Published:** 2015-08-14

**Authors:** Andrew J. Taylor, Daniel H. Paris, Paul N. Newton

**Affiliations:** 1 Lao Oxford-Mahosot Hospital-Wellcome Trust Research Unit (LOMWRU), Microbiology Laboratory, Mahosot Hospital, Vientiane, Laos; 2 Centre for Tropical Medicine and Global Health, Nuffield Department of Clinical Medicine, Churchill Hospital, University of Oxford, Oxford, United Kingdom; 3 Mahidol–Oxford Research Unit (MORU), Faculty of Tropical Medicine, Mahidol University, Bangkok, Thailand; University of Texas Medical Branch, UNITED STATES

## Abstract

**Background:**

Scrub typhus, a bacterial infection caused by *Orientia tsutsugamushi*, is increasingly recognized as an important cause of fever in Asia, with an estimated one million infections occurring each year. Limited access to health care and the disease’s non-specific symptoms mean that many patients are undiagnosed and untreated, but the mortality from untreated scrub typhus is unknown. This review systematically summarizes the literature on the untreated mortality from scrub typhus and disease outcomes.

**Methodology/Principal Findings:**

A literature search was performed to identify patient series containing untreated patients. Patients were included if they were symptomatic and had a clinical or laboratory diagnosis of scrub typhus and excluded if they were treated with antibiotics. The primary outcome was mortality from untreated scrub typhus and secondary outcomes were total days of fever, clinical symptoms, and laboratory results. A total of 76 studies containing 89 patient series and 19,644 patients were included in the final analysis. The median mortality of all patient series was 6.0% with a wide range (min-max) of 0–70%. Many studies used clinical diagnosis alone and had incomplete data on secondary outcomes. Mortality varied by location and increased with age and in patients with myocarditis, delirium, pneumonitis, or signs of hemorrhage, but not according to sex or the presence of an eschar or meningitis. Duration of fever was shown to be long (median 14.4 days Range (9–19)).

**Conclusions:**

Results show that the untreated mortality from scrub typhus appears lower than previously reported estimates. More data are required to clarify mortality according to location and host factors, clinical syndromes including myocarditis and central nervous system disease, and in vulnerable mother-child populations. Increased surveillance and improved access to diagnostic tests are required to accurately estimate the untreated mortality of scrub typhus. This information would facilitate reliable quantification of DALYs and guide empirical treatment strategies.

## Author Summary

Scrub typhus is a common cause of fever in rural Asia where there is limited access to healthcare, diagnostics, and treatment. It is thought that up to 1 million cases occur per year, but the disease is difficult to differentiate clinically from other infections, such as leptospirosis and dengue, meaning that many infections go undiagnosed and untreated. This review systematically summarizes the literature on the untreated mortality of scrub typhus and describes the outcome from untreated disease. Many articles had incomplete data and relied on a clinical diagnosis of disease, but results showed that scrub typhus is associated with a lower mortality than previously described, at approximately 6%, ranging from 0–70%. Further information is required to clarify the mortality according to location, host factors, and clinical syndromes. More widespread access to medical care, coupled with the increased use of affordable and accurate rapid tests, would improve the diagnosis and treatment of scrub typhus. Increased surveillance and investigation into differences in strain virulence by region are required to reliably quantify DALYs and the disease burden from scrub typhus, and to guide empirical treatment strategies.

## Introduction

Scrub typhus is caused by infection with the intracellular bacteria *Orientia tsutsugamushi*, which is transmitted by the bite of larval trombiculid mites. The disease is known to occur throughout Asia, but recent evidence suggest that its range may be larger, with case reports in Africa [1,2], Chile [3] and a new related species, *O*. *chuto*, described in the Middle East [4]. In Southeastern Asia, it is thought that up to 1 million cases occur per year [5,6], and a significant proportion of hospital admissions for acute undifferentiated fever have been shown to be attributable to scrub typhus [5,7]. The disease is most common in rural areas, where there is limited access to healthcare, diagnostics and treatment, and is difficult to differentiate from other infections, such as leptospirosis and dengue, on a clinical basis alone. Eschar, a diagnostic clue, is not always present, while access to rapid tests is not widespread and the tests are limited by low sensitivity, especially early in the disease course [8]. Laboratory diagnosis with the gold standard Indirect Immunofluorescence Assay (IFA) is expensive and impractical in rural areas. Effective treatment with doxycycline or azithromycin is available, but evidence of resistance to doxycycline in Northern Thailand [9] raises concern of undiscovered resistance elsewhere [10].

Due to these factors, many scrub typhus infections remain undiagnosed or untreated, but the outcomes from untreated infections are unknown. Current estimates of the untreated mortality from scrub typhus are unclear, with those from before the age of antibiotic therapy as high as 40–45% [11,12]. Estimating the true mortality, however, is challenging as disease severity is thought to vary according to regional strains [13,14], infectious dose [15,16], patient age, and comorbidities [17]. This review aims to estimate the untreated mortality from symptomatic scrub typhus through a comprehensive review of literature. Such understanding will improve our current understanding of the untreated mortality from scrub typhus and help estimate the burden of disease.

## Methods

### Eligibility criteria

Literature was reviewed for patient series containing untreated patients with scrub typhus. Included patients were of any age or sex and had fever and clinical symptoms consistent with scrub typhus, with or without a confirmed laboratory diagnosis through culture, inoculation of laboratory animals, serological tests, or the Polymerase Chain Reaction (PCR). Patients were defined as untreated if they had not been treated with antibiotics or convalescent serum, and had not been vaccinated against scrub typhus. Patients admitted to hospital for supportive treatment, including intravenous fluids, or Intensive Care Units (ICU), were included in the analysis. Clinical diagnosis was defined per individual patient series and included patients with no eschar, but patients with evidence suggestive of an alternative diagnosis were excluded. The primary outcome of the analysis was mortality from untreated scrub typhus. Secondary outcomes were total days of fever, clinical symptoms, and laboratory results where available. All study designs describing the untreated mortality of scrub typhus were included. Patient series with fewer than 10 patients were excluded to reduce selection bias. All published papers regardless of year of publication were included in the search. Journals in European languages (English, French, German, Dutch) were included but those in other languages were excluded. This review followed the PRISMA statement for systematic reviews (S1 Checklist).

### Information sources, search strategy and study selection

Articles were identified through electronic resources, through scanning of reference lists of relevant articles, and from library index catalogues. The electronic search was performed using Ovid MEDLINE (1946—Present), Embase Classic (1947 –Present), and Global Health (1910 –Present) on 28^th^ July 2014. The electronic databases were searched using “scrub typhus or *Orientia tsutsugamushi* or *Rickettsia tsutsugamushi* or Orientia tsu* or Akamushi disease or Japanese river fever or mite typhus or tropical typhus or tsutsugamushi disease” and a second search for “mortality or death”. (S1–S3 Figs). Duplicate search results were removed using Mendeley (2008–14 Mendeley Ltd, Version 1.12.1). Authors were not contacted regarding further information due to the age of many of the articles, no unpublished literature was obtained, and abstracts were not included if a full article was unobtainable. AJT reviewed abstracts and titles from all search results to assess eligibility and if there was doubt as to the relevance of the article from the abstract and title alone, the full article was obtained and then conferred with the other authors.

### Data extraction, methodological assessment, summary measures and planned method of analysis

AJT extracted data for geographical location, year of study, study design, number of patients, patient demographics, clinical symptoms and signs, laboratory results, diagnostic test and mortality, noting missing data. Patient series were extracted separately if an article contained more than one patient series. Each patient series was reviewed with respect to other articles and duplicates excluded. Untreated patients in partially treated series were extracted when possible, but excluded if the paper did not specifically express outcomes for untreated patients. Articles not in English were translated using Google translate where necessary. A standardized form was created to assess bias in patient series (S1 Table). Four criteria were assessed on a 3-point grade scale: patient selection, diagnostic test, patient information and outcomes. Diagnosis was considered Grade I if *O*. *tsutsugamushi* were cultured or there was a 4-fold rise in titre on IFA, Grade II if there was a single high titre on IFA, or Weil-Felix (OXK) test was positive for all included patients, or Grade III if there was a clinical diagnosis alone or no laboratory diagnosis for all included patients. The primary outcome of the review was the median mortality (range) across all patient series and termed the “median series mortality”. Secondary outcomes were measured as the median value across patient series unless otherwise stated. The chi-squared test was used to compare overall secondary outcomes when appropriate. Data was mapped using an image from NASA—Visible Earth.

## Results

**Fig 1:**
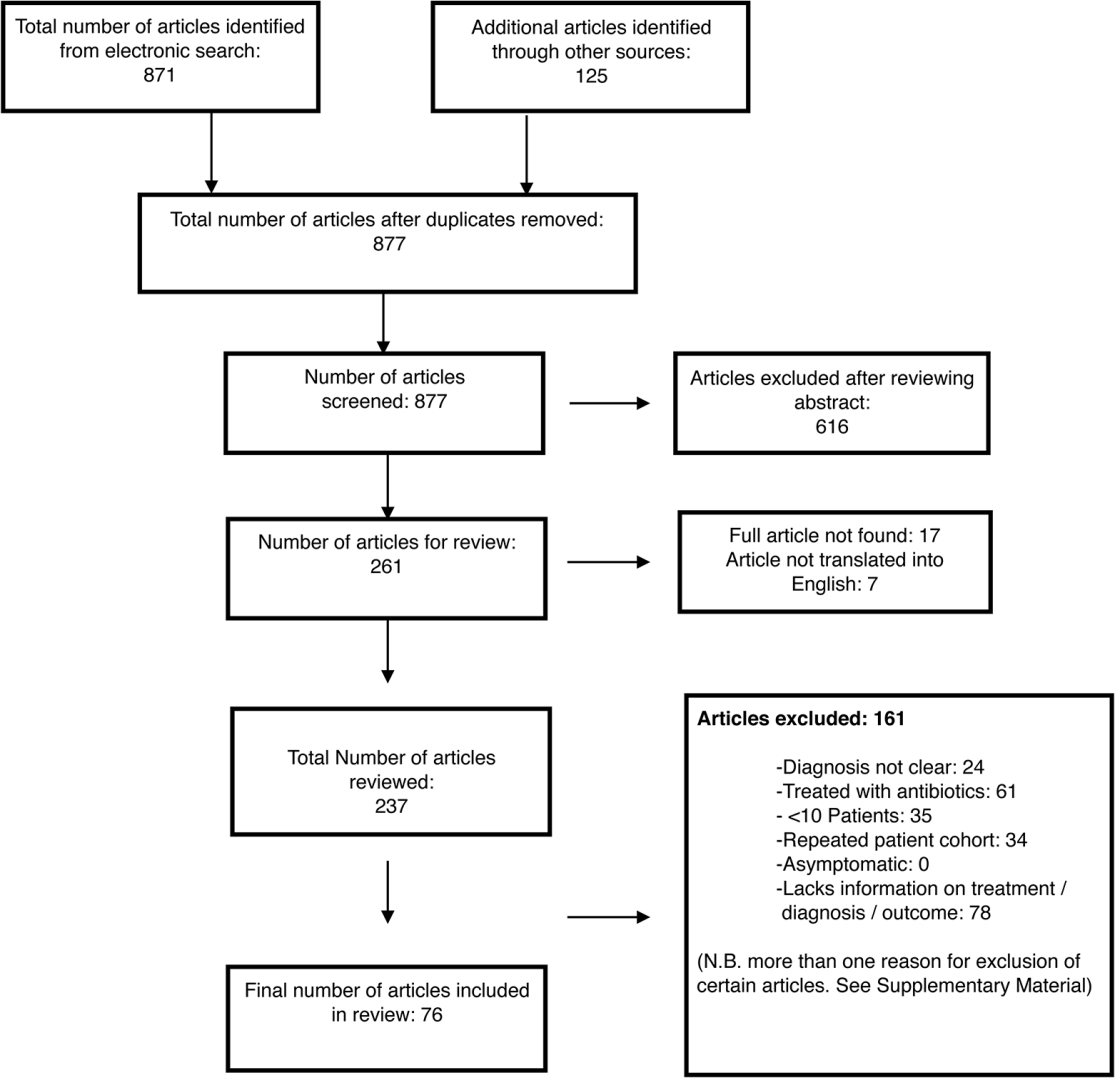
Flow chart showing selection of studies for the review.

Seventy-six articles, containing 89 patient series, and a total of 20,307 patients were identified for inclusion (Fig 1). Within these studies, 663 patients had an alternative or unclear diagnosis, or were included in other series, and were excluded, giving a total of 19,644 patients in the final review. Seventeen articles were excluded as the full text could not be obtained (S2 Table) and 7 studies were excluded, as they were not translated into English. Full details of reasons for article inclusion and exclusion are displayed in Fig 1 and S3 Table.

### Study characteristics

**Fig 2:**
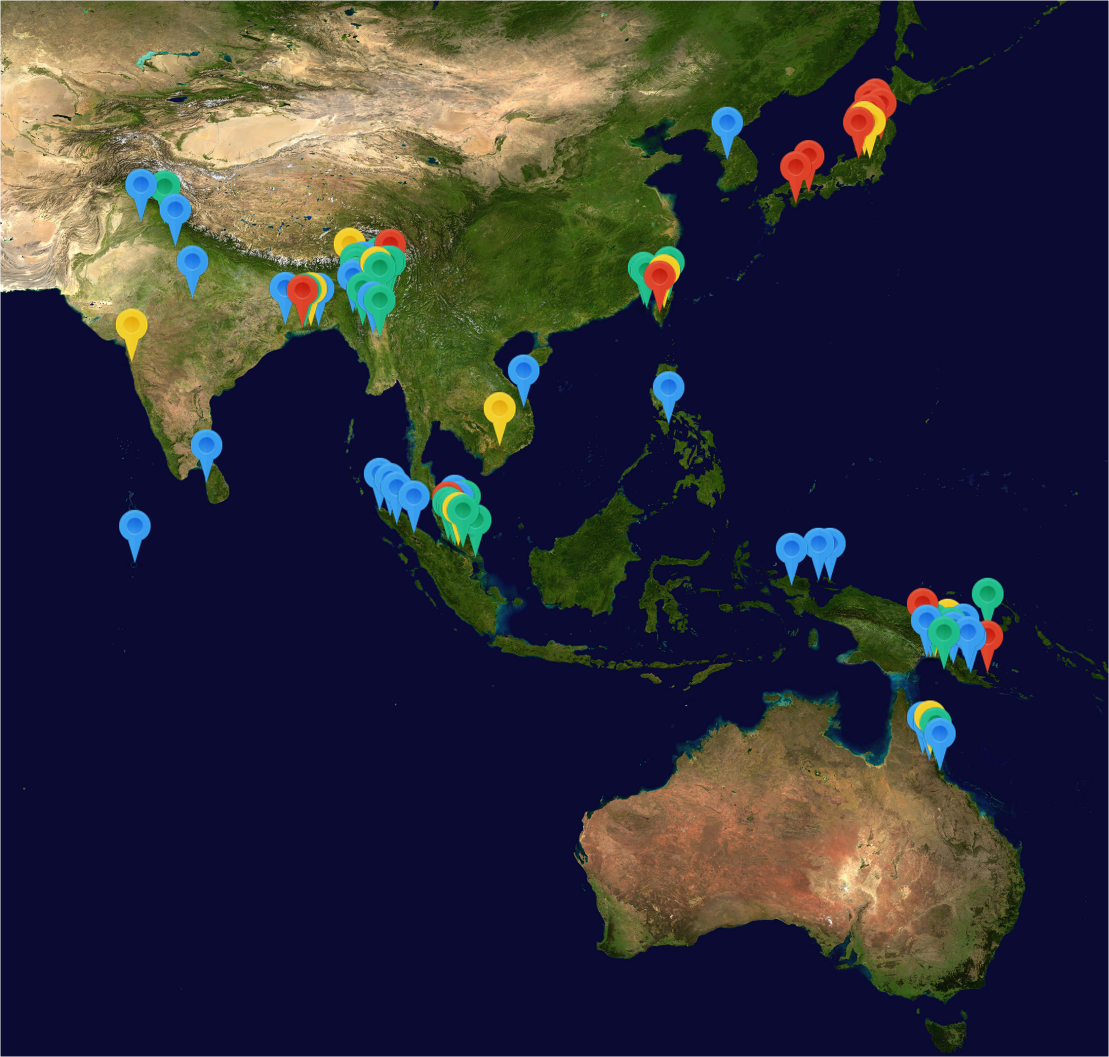
Location of included patient series. Series are colour coded by median series mortality. Blue = 0–5%. Green = 5–10%, Yellow = 10–20% and Red = >20%. Map image: NASA–Visible Earth

Details of included articles are displayed in S4 Table. Articles were published between 1878 and 2008 and size of patient series ranged from 10 to 1,522 patients. Sixty-seven articles were in English, 4 in German, 3 in Dutch and 2 in French. Nine patient series were prospective cohort series, 2 controlled trials, 75 retrospective series, and 3 summaries of case reports. All studies were hospital based with twenty-eight undertaken in the Indian Subcontinent (India, Myanmar, Sri Lanka and Pakistan), 18 in New Guinea, 11 in Japan, 11 in Malaysia or Singapore, 5 in Australia, 5 in Taiwan, 4 in Indonesia (excluding New Guinea), 2 in Cambodia or Vietnam, 1 in the Philippines and 1 in Korea (Fig 2). Three further patient series were infected using inoculation of experimental strains of *Orientia tsutsugamushi*. Individual patient series were assessed for methodological quality using a data extraction sheet designed for this review. A summary of bias within each study is displayed in S5 Table and more detailed information is contained in S6 Table. Many of the patient series had a high level of bias with significant missing data. There was a high risk of bias in diagnosis in 75.3% (67/89) of studies, due to reliance on clinical diagnosis alone in these papers, while only 4.5% (5/89) papers had a confirmed diagnosis through culture, paired serology or inoculation of *O*. *tsutsugamushi*.

### Outcomes

**Fig 3:**
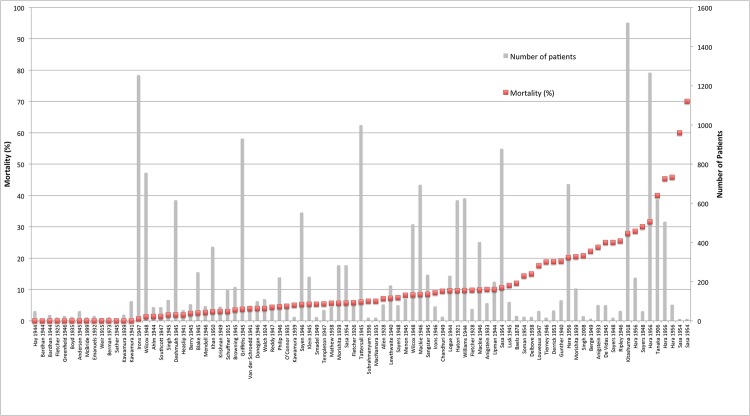
Mortality (%) across patient series. Median series mortality is displayed as red squares and number of patients in each series as grey bars.

Patient mortality data were available for 89/89 patient series for a total of 19,644 patients. Median series mortality was 6.0% with a wide range across series (0–70%). Overall 12.7% (2,488/19,644) of patients died (Fig 3 and Table 1). Information on individual secondary outcomes was available in as few as 3 of 89 reports and up to 65 of 89 studies for each outcome. The majority of included studies reported a high median incidence of headache (100% (71.0–100)) and lymphadenopathy (84.7% (20.0–100%)), while conjunctival congestion (69.3% (10.2–100%)), myalgia (56.1% (2.2–100%)) and cough (50% (5.1–100%)) were reported in a significant proportion of patients. Haemorrhagic symptoms ranged from epistaxis to more severe bleeds and the definition of pneumonitis varied between studies, often being diagnosed on clinical grounds. Pneumonia, as confirmed radiologically, was defined separately. Definition of myocarditis varied between studies and was often on a clinical basis alone. No included patients were documented as receiving treatment on ICU, mechanical ventilation, or vasopressor support.

**Table 1:** Demographics, clinical symptoms and laboratory data across patient series.

Criteria	Number of Patient Series (from 89)	Number / total number of patients	Median Value Across Patient Series (range)
Age	14	1684	**29 years** (22–37) (22.0–34.5)
Sex	65	12,171/13,369	**100% Male** (51.3–100)
**Symptoms**			
Duration of Fever	25	3927	**14.4 days** (9–19)
Headache	29	3,805/4,251	**100%** (71.0–100)
Conjunctival Congestion	22	1,860/2,765	**69.3%** (10.2–100)
Myalgia	13	1,051/2,417	**56.1%** (2.2–100)
Cough	28	1,706/3,869	**50%** (5.1–100)
Pharyngitis	12	598/1,301	**37.0%** (1.9–100)
Deafness	23	756/4,020	**25.0%** (0.0–74.4)
Abdominal Pain	11	155/825	**14.3%** (2.8–80.4)
Delirium	24	293/2,983	**12.6%** (0.0–56.9)
Nausea or vomiting	10	131/1,738	**8.4%** (2.4–43.8)
Bleeding (Inc. epistaxis)	14	150/1,828	**6.2%** (1.0–55.6)
Meningitis	19	141/2,925	**5.5%** (0.0–15.8)
**Signs**			
Lymphadenopathy	41	6,289/7,534	**84.7%** (20.0–100)
Rash	51	3,526/7078	**44.9%** (0–100)
Eschar	57	4,810/9,017	**37.7%** (0–100%)
Splenomegaly	25	1,103/3,051	**35.0%** (0.0–95.5)
Pneumonitis	27	1,655/4,673	**33.9%** (2.3–68.0)
Hepatomegaly	7	200/836	**14.3%** (6.4–93.5)
Pneumonia	28	286/3,907	**7.8%** (1.0–38.1)
Myocarditis	6	58/906	**4.3%** (1.0–39.2)
Jaundice	6	120/1,823	**1.9%** (0.0–3.1)
**Blood Laboratory Results**			
White blood cells per microlitre	18	1,180	**6,450** (5,000–12,000)
“Raised Urea” *	3	37/176	**18.9** (0.0–24.8)

* *Definition of raised urea ranged from >8*.*9mmol/L to 21*.*4mmol/L*.

**Fig 4:**
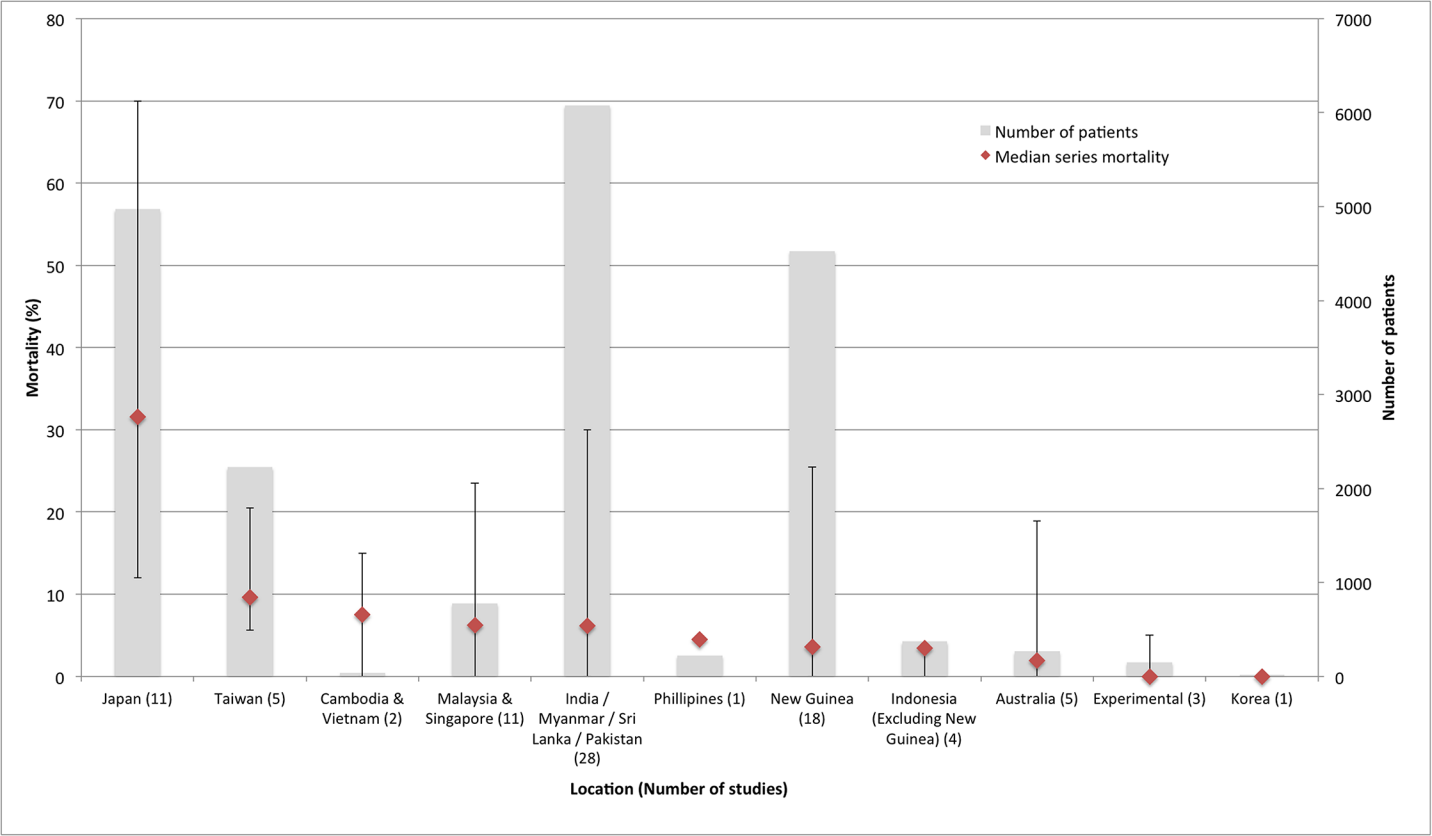
Descending median series mortality by location. Mortality is displayed in red with error bars displaying maximum and minimum values across studies. Grey bars indicate total number of patients for included studies in each location. Number of studies in each region is displayed in brackets after each location label.

Median series mortality was higher in Japan at 31.6% (12–70%) than in other regions, where median patient series mortality was below 10% (0–30%) (Fig 4). Data on mortality by year of patient series were present for all patient series (89/89) with a wide range in mortality over time but no overall discernable trend in mortality according to year. Mortality varied according to study design with median series mortality 12.1% (5.3–18.8%) in 2 controlled trials, 6.25% (0–23.5%) in 9 prospective case series, 5.7% (0–45.8%) in 75 retrospective case series, and 60% (19–70%) in 3 patient series summarising case reports. Median series mortality in 5 patient series with a Grade I diagnosis (mouse inoculation with *O*. *tsutsugamushi*, *in vitro* isolation of *O*. *tsutsugamushi* or use of paired IFA sera) was 0% (0–14.3%), in 17 series with a Grade II diagnosis (single high titre OXK) 3.0% (0–23.5%), and in 67 studies with a Grade III diagnosis (clinical diagnosis alone for some patients) 8.3% (0–70%). No studies used PCR to diagnose infection.

### Patient characteristics

Data on mortality by age were included in 15/89 patient series for 3,879 patients (Table 2) and showed a trend towards increasing mortality with age. Overall mortality in patients under 30 was 10.7% (241/2261), compared to overall mortality of 21.3% (344/1618) in all patients over 30. Information on mortality by sex was available in 60/89 series for male patients and 14/89 series for female patients. Median series mortality was 4.9% (range 0–30%) with overall mortality 6.9% (851/12,273 deaths) in males compared to median series mortality 1.4% (range 0–32.2%) and overall mortality 23.5% (181/771 deaths) in female patients. The majority of included patients in this review were male and sample sizes for series including female patients were often small, with many series reporting less than 10 patients. One series of 519 female patients [18] reported 179 deaths (34.5%) and had a large influence on the overall female mortality.

**Table 2:** Patient mortality by age.

Age	Number of Patient Series	Overall % Mortality (Number of deaths / Total number of patients across series)	Median series mortality (%) (Range (%)
1–10	6	11.1 (20/180)	1.8 (0–33.3)
11–20	12	11.0 (72/653)	1.6 (0–25)
21–30	12	12.0 (109/905)	0.0 (0–66.7)
31–40	10	13.9 (101/729)	0.0 (0–31.6)
41–50	11	18.7 (74/396)	0.0 (0–36.2)
51–60	7	45.6 (92/242)	5.9 (0–47.6)
61+	4	59.8 (52/87)	29.4 (0–100)

### Mortality according to patient symptoms or signs

Data on patient mortality according to patient symptoms or signs were not available from all studies due to limitations in data collection and description in many studies. Available data (Table 3) showed that the presence of myocarditis, haemorrhagic symptoms, delirium and pulmonary symptoms were associated with increased overall mortality, but the presence of eschar or meningitis were not.

**Table 3:** Patient mortality according to presence or absence of symptoms or signs.

Symptoms	Symptoms present			Symptoms not present			Difference in overall
	Number series present	% Overall mortality (Deaths/patient number)	Median series mortality (range)	Number series not present	% Overall mortality (Deaths / patient number)	Median series mortality (range)	mortality (Chi squared test)
**Myocarditis**	4	24.0 (12/50)	34.2 (8.3–66.7)	4	4.0 (14/346)	3.2 (0.0–6.8)	**p < 0.001**
**Haemorrhage**	8	23.7 (23/97)	0.0 (0.0–100)	8	1.2 (6/520)	0.0 (0.0–3.6)	**p < 0.001**
**Delirium**	11	16.9 (20/118)	0.0 (0.0–60.0)	13	2.0 (39/1916)	0.0 (0.0–9.5)	**p < 0.001**
**Meningitis**	10	8.6 (13/151)	6.3 (0.0–100)	11	6.1 (63/1040)	2.0 (0.0–23.9)	**p = 0.23**
**Pneumonitis**	18	7.2 (56/782)	1.1 (0.0–30.0)	18	0.3 (6/1794)	0.0 (0.0–8.3)	**p < 0.001**
**Eschar**	19	5.8 (108/1861)	2.1 (0.0–25)	27	4.7 (68/1,460)	0.0 (0.0–18.9)	**p = 0.14**

## Discussion

This review is the first to systematically assess the untreated mortality from scrub typhus. The median series mortality in untreated patients was shown to 6.0%, but there was a wide range (0–70%) in series mortality reflecting the broad inclusion criteria, differences in study design, and the clinical spectrum of disease. There was a high degree of bias within individual studies, due to the frequent reliance on clinical diagnosis alone, and the shortcomings of the available data. Despite these limitations, results showed that median series mortality was lower than previous conservative estimates of 10% or more [5,6,19,20], but that patients had a long median duration of fever of 14.4 days (9–19), suggesting that scrub typhus has a high overall burden of morbidity

The majority of studies included in this review were undertaken before the introduction of antibiotics in the late 1940s and based on a clinical diagnosis of scrub typhus alone. Only 5 studies, containing 191 patients, reported a grade I diagnosis for all included patients, and showed an upper mortality of untreated scrub typhus of 14.3%. In studies with a clinical diagnosis alone (Grade III diagnosis), untreated mortality reached 70%. Mortality over 30% was only reported in early 20^th^ century Japanese case series [21–23], which had a high degree of bias due to clinical diagnosis and reliance on a case notification system. More accurate diagnostics are required to give a more precise estimate of the true mortality from scrub typhus.

The broad range in mortality demonstrated in this review is consistent with previous findings [20], but the reasons for this variability are not understood and may be dependent on location, strain, and host factors. Studies from the Second World War [24], and more recent research [19], suggest that mortality is location and outbreak specific. Differences in strain virulence have been demonstrated experimentally in mice where inoculation of *O*. *tsutsugamushi* strains isolated from patients from series with a low mortality caused a low mortality, while those from outbreaks with a high mortality caused a higher mortality [13]. Previous pathogen exposure may also play a role in the incidence and mortality from scrub typhus. Several patient series [25–28] have reported a lower case incidence and fatality in native populations than in immigrant populations with no history of previous exposure. This suggests a role of immunity in reducing mortality from scrub typhus in the pre-exposed, although previous vaccine work has showed that immunity is complex and strain dependent [29]. A recent study has shown that differences in the initial inoculated bacterial load [30] may also influence mortality.

Patient factors are thought to influence mortality. In this study, mortality increased across age strata, with a higher overall mortality in patients over 30 than those under 30 years, and the highest mortality in patients over 60 years, although patient numbers were small and the diagnosis often clinical. Increasing mortality with age is likely to be influenced by comorbidities, which increase with age, and immunosenescence. Co-infection or physical exhaustion [17] may also lead to a higher mortality, with a reported mortality of 25% in 150 post-combat soldiers co-infected with dysentery or malaria in Papua New Guinea [31]. It unknown whether gender influences mortality and in this study the majority of patients were male (88.6%), mostly Second World War soldiers, so it is not possible to draw strong conclusions on differences in mortality between sexes. Only two pregnant women were included in this review but both had abortions and further work is required to better understand the mortality of scrub typhus in women, during pregnancy, and in neonates [32].

The presence of specific clinical symptoms may predict increased mortality, although evidence from this study was mostly based on data from patients with a clinical diagnosis. A significantly higher overall mortality was shown in patients with myocarditis and haemorrhagic, neurological or pulmonary symptoms, but not in those with meningitis or an eschar. Myocarditis was diagnosed clinically in the majority of studies in this series, which is often inaccurate, but is thought to be a rare but serious complication of scrub typhus with a high mortality [14,20], and future investigation into its importance is merited. Similarly, haemorrhagic complications were uncommon, but associated with significant mortality [33]. Delirium was associated with increased mortality, although meningitis was not, which contrasts with more recent studies which have suggested significant mortality in those patients with meningitis [34]. Interestingly there was no significant difference in patient mortality according to the presence of eschar. This is a key clinical sign as patients without an eschar are more likely to be misdiagnosed and remain untreated, and suggests that disease in this group is of equal severity. Other clinical finding of note were the longer duration of fever in patients (median 14.4 days (9–19)) than those seen in recent studies [34,35], suggesting significant morbidity from the disease. Incidence of conjunctival suffusion (median 69.3% (10.2–100%)) and lymphadenopathy (84.7% (20–100%)) was shown to be high, which could cause diagnostic confusion with other infections such as leptospirosis or dengue.

All studies included in this review recruited patients in hospitals. A significant but unknown proportion of patients with scrub typhus, however, do not seek appropriate medical care, or attend facilities where no reliable diagnostics are available, so were not diagnosed and included in this review. Of note, no patients in this study were treated on an ICU, which reflects the age of many of the studies and the use of antibiotic treatment in recent patients treated on ICU. Study design also affected outcome as a higher median mortality was shown in articles summarizing case reports 60% (range 19–70%), which were likely to select for more severe disease, than in other study designs. The true untreated mortality from scrub typhus may therefore be lower than previously thought, with data from this review suggesting that ~ 94% of patients survive a natural untreated disease course. Further work is needed to clarify the social and economic impact of infection with scrub typhus as its low mortality but widespread morbidity are likely to have a high burden of DALYs and a high economic impact.

There are several limitations to this study, which may limit the applicability of results. Firstly, publication bias is likely due to the limitations of electronic searches for older literature, exclusion of non-European language papers, and the use of reference lists to identify relevant articles. Secondly, included patient series may not have been representative of all patients with scrub typhus. Many series relied on clinical diagnosis of the disease or retrospective case series, which are often less accurate, while several studies were excluded, as there was no separately recorded outcome for untreated patients. Thirdly, the high degree of bias across studies due to the age and heterogeneity of studies, the lack of standardization of study design or clearly defined methodology, the significant proportion of missing data, and the removal of treated patients from untreated cohorts, reduced reliability of the study findings.

### Conclusions

Results of this study show that mortality from scrub typhus varies greatly, but is lower than commonly reported estimates. Morbidity from the disease however, is significant, due to the prolonged duration of fever in scrub typhus (median 14.4 days (range 9–19)). Further work is required to clarify mortality according to location and host factors, and patient symptoms including myocarditis, central nervous system disease and in vulnerable mother-child populations. Results of this study suggest that further investigation into differences in strain virulence by region are required to reliably quantify DALYs, measure the disease burden from scrub typhus, and to guide empirical treatment strategies. More widespread access to medical care, coupled with the increased use of affordable and accurate rapid tests, is required to improve diagnosis and treatment of these easily treatable infections.

## Supporting Information

S1 Checklist PRISMA (Preferred Reporting Items for Systematic Reviews and Meta-Analyses) checklist. (PDF)

S1 Fig Search results for Ovid MEDLINE database, 28^th^ July 2014. (TIF)

S2 Fig Search results for Embase Classic + Embase database, 28^th^ July 2014. (TIF)

S3 Fig Search results for Global Health database, 28^th^ July 2014. (TIF)

S1 Table Assessment of bias within studies. A previous systematic review, which had assessed clinical outcome from disease, was used to guide assessment of study design and patient selection, while a grading system for diagnostics was adapted from WHO criteria for diagnosis of leptospirosis and Phommasone *et al*.
(DOCX)

S2 Table Articles not obtained for inclusion in the review. (DOCX)

S3 Table Reasons for article exclusion. (DOCX)

S4 Table Characteristics of included studies, arranged chronologically within each region. When studies contained more than one patient series, each series was displayed separately. All available data are included, but if data were not available it is indicated by a “-“. Percentage and (number/total number of patients) are quoted, but if no patient number was quoted then a “NA” is used.
(DOCX)

S5 Table Risk of bias within studies. Red reports high risk of bias, yellow medium risk and green low risk of bias.
(DOCX)

S6 Table Further information on bias within studies. (DOCX)
