# Supplementary figures and images for: A Systematic Review of Mortality from Untreated Scrub Typhus (Orientia tsutsugamushi)
Source: PLoS Negl Trop Dis. 2015 Aug 14;9(8):e0003971. doi: 10.1371/journal.pntd.0003971 (PMC4537241; doi:10.1371/journal.pntd.0003971)

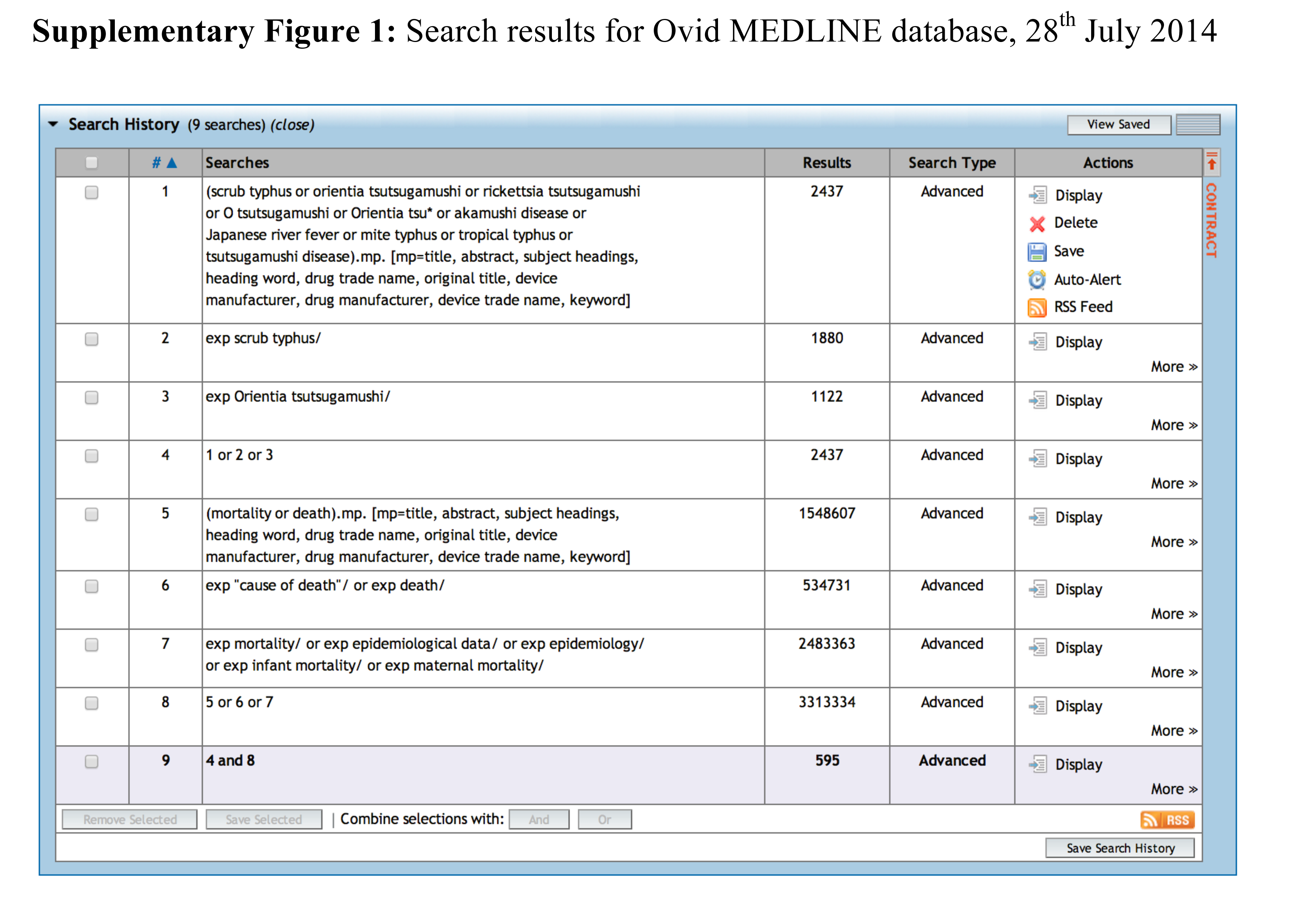

Supplement: S1 Fig — (TIF) [file pntd.0003971.s002.tif]

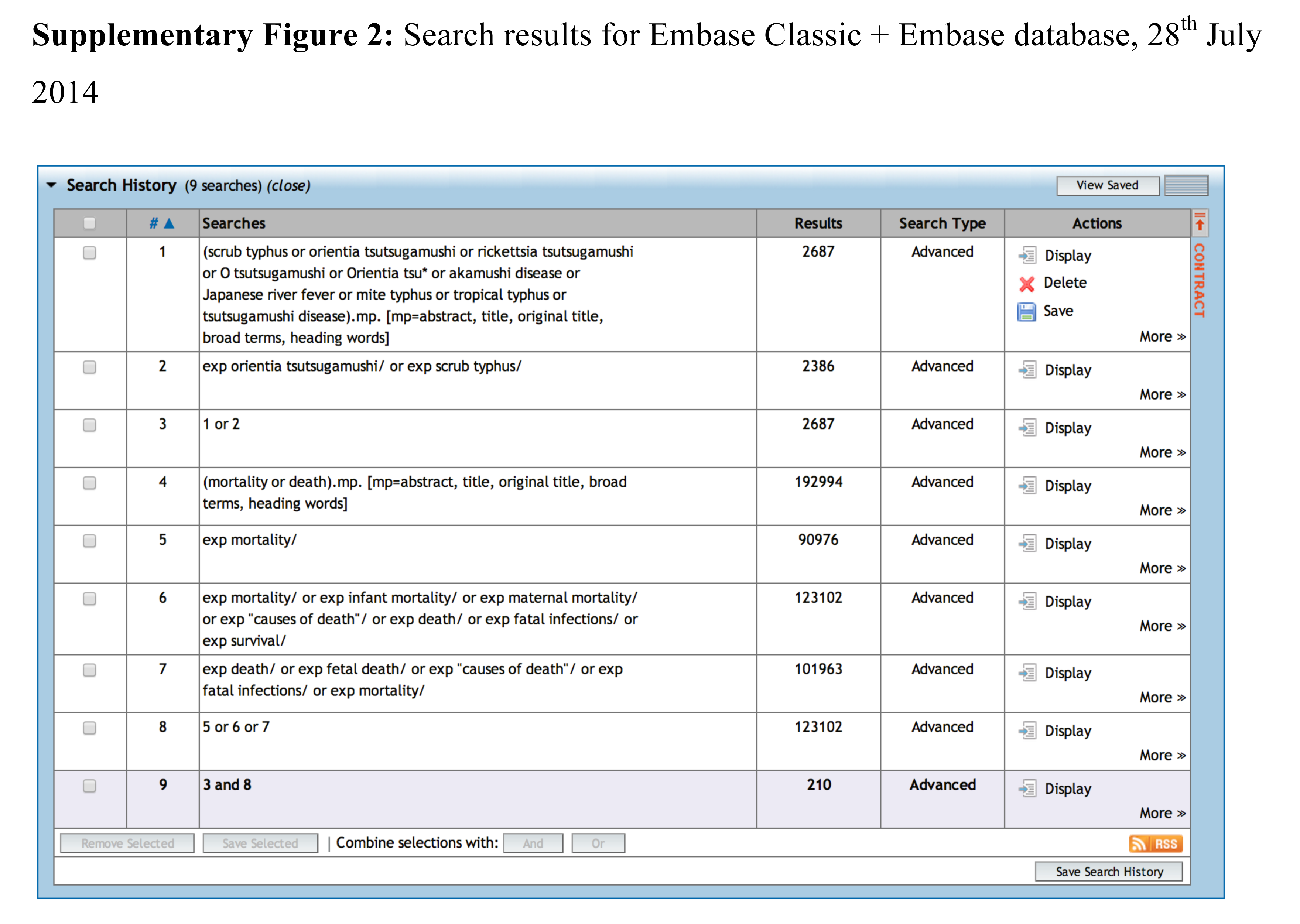

Supplement: S2 Fig — (TIF) [file pntd.0003971.s003.tif]

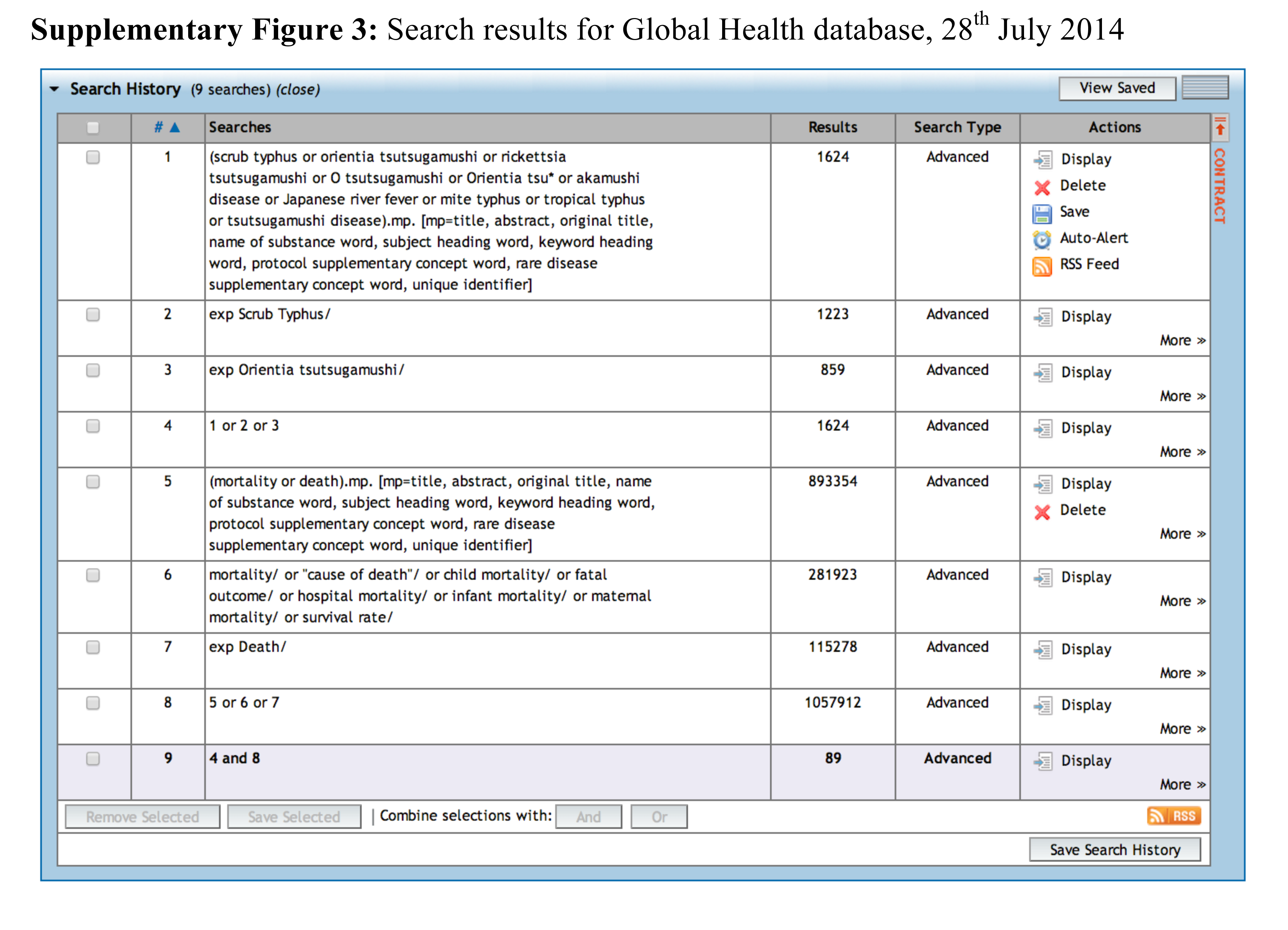

Supplement: S3 Fig — (TIF) [file pntd.0003971.s004.tif]
