# Supplementary material for: A Systematic Review of Mortality from Untreated Scrub Typhus (Orientia tsutsugamushi)
Source: PLoS Negl Trop Dis. 2015 Aug 14;9(8):e0003971. doi: 10.1371/journal.pntd.0003971 (PMC4537241; doi:10.1371/journal.pntd.0003971)
Supplement: S1 Table — A previous systematic review, which had assessed clinical outcome from disease, was used to guide assessment of study design and patient selection, while a grading system for diagnostics was adapted from WHO criteria for diagnosis of leptospirosis and Phommasone et al. (DOCX) [file pntd.0003971.s005.docx]

**Supplementary Figure 1: Assessment of bias within studies.** A previous systematic review which had assessed clinical outcome from disease was used to guide assessment of study design and patient selection (1). A grading system for diagnostics was adapted from WHO criteria for diagnosis of leptospirosis (2) and Phommasone *et al.* (3).

1. Borghouts JA, Koes BW, Bouter LM. The clinical course and prognostic factors of non-specific neck pain: a systematic review. Pain. 1998 Jul;77(1):1–13.

2. World Health Organisation. Report of the First Meeting of the Leptospirosis Burden Epidemiology Reference Group, Geneva. WHO. Geneva; 2010;1–34.

3. Phommasone K, Paris DH, Anantatat T, Castonguay-Vanier J, Keomany S, Souvannasing P, et al. Concurrent Infection with murine typhus and scrub typhus in southern Laos--the mixed and the unmixed. PLoS Negl Trop Dis. 2013 Jan;7(8):e2163.

| **Category** | **Grade** | **Criteria** |
| --- | --- | --- |
| **Patient Selection** | Grade I | - Prospective, consecutive patient case series with no inappropriate exclusions. |
|  | Grade II | -Prospective, non-consecutive case series  -Retrospective case series  -Reference laboratory series |
|  | Grade III | -Exclusion of patients (i.e. most unwell patients treated) likely to significantly affect outcome  -Summary of case reports from literature. |
|  |  |  |
| **Diagnostic Test** | Grade I | -Detection of Rickettsia though cell culture or animal inoculation.  -IFA Serological diagnosis with either sero-conversion or fourfold antibody response |
|  | Grade II | -Single high OX-K titre of ≥1:100 for all samples  -Single high IFA titre >1:50 for all samples |
|  | Grade III | -Single high OX-K titre but titre non-significant for all patients  -No record of significant OX-K titre  -Clinical diagnosis |
|  |  |  |
| **Missing information** | Grade I | - No information missing |
|  | Grade II | - Information missing on **1 or 2** of age / sex / adenopathy / duration of fever / eschar / complications |
|  | Grade III | - Information missing on **3** or more of age / sex / adenopathy / duration of fever / eschar / complications |
|  |  |  |
| **Outcome** | Grade I | -Known for all patients. No patients excluded |
|  | Grade II | -Outcome not known for all patients |
|  | Grade III | -Outcome not included for all patients in series due to treatment |
