# Supplementary material for: A Systematic Review of Mortality from Untreated Scrub Typhus (Orientia tsutsugamushi)
Source: PLoS Negl Trop Dis. 2015 Aug 14;9(8):e0003971. doi: 10.1371/journal.pntd.0003971 (PMC4537241; doi:10.1371/journal.pntd.0003971)
Supplement: S2 Table — (DOCX) [file pntd.0003971.s006.docx]

**Supplementary Table 2: Articles not obtained for inclusion in the review**

| Bowes BT. Tick Borne Typhus. Proceedings of the Conference of Medical Specialists of Eastern Army. 1943. p. 68. |
| --- |
| Faust EC. The possible incidence of river fever (tsutsugamushi disease) in the central Yangtze valley. China Med J. 1923;37:979–87. |
| Kawahigasi K. Experiences with tsutsugamushi fever espescially with the so-called atypical cases. Taiwan Igakkai Zasshi = J Med Assoc Formosa. 1941;40:355–67. |
| Kawamura R, Imagawa Y. Ein neues Phanomen post-mortaler Proliferation der Rickettsien bei der Tsutsugamushi-Krankheit. Zentralblatt fur Bakteriol. 1932;125:304–12. |
| Kimura R. Untersuchungen uber der Erreger der Tsutsugamushi-Krankheit in der Gewebekulturen. Trans Soc Pathol Japan. 1932;22:668–70. |
| Ko T. Clinical Observations on 100 Cases of Tsutsugamushi Disease. Taiwan Igakkai Zasshi = J Med Assoc Formosa. 1934;33(4 (349)):51. |
| Lewthwaite R. Clinical and Epidemiological Observations on Tropical Typhus in the Federated Malay States. Bull Inst Med Res, Fed Malay States. 1930;(1):42 – pp. |
| Matsumoto T. On tsutsuhamushi disease in Middle Formosa. Taiwan Igakkai Zasshi = J Med Assoc Formosa. 1930;303:632–8. |
| Morishita K. The distribution and epidemiology of tsutsugamushi disease in Formosa. Taiwan sotokufu chuokenkyusho eiseibugyoho. 1934;2216:76. |
| Morishita K. Tsutsugamushi disease o Pescadores. Tokyo Iji-Shinshi = Tokyo Med News. 1939;64(4). |
| Nagoyo M. Ueber die Tsutsugamushi-Krankheit. Tokyo Igakkwai Zasshi = J Tokyo Med Soc. 1915;29:1439–62. |
| Norman JP. Scrub Typhus with special reference to a Yearly Outbreak occurring on Cherldeo T. E. Proc Ann Gen Meet Assam " Branch Brit Med Ass, Cinnamara, 12th-15th March, 1953. Calcutta; 1953;17–22. |
| Tanaka K, Kaiwa J, Teramura S, Kagaya J. Beitrage zur japanischen Kedani-Krankheit. Zentralblatt fur Bakteriol. 1930;116:353. |
| Wolff JW. “Tropical typhus” een viektyphus-achtige ziekte. Geneeskd Tijdschr voor Ned. 1929;69:429–61. |
| Yang Z, Yu X, Liu Y. Studies on clinical epidemiology of Tsutsugamushi disease of the autumn-winter type in the easten suburbs of Jinan. Zhonghua Liu Xing Bing Xue Za Zhi. 1997;18(4):233–5. |
| Yeomans A. Progress in rickettsial disease therapy. With special reference to para-aminobenzoic acid. J Lancet. A. Yeomans, U.S.A. Typhus Commission, War Department, Washington, DC, United States; 1947;67(2):60–3. |
| Colonial Research 1955-1956. Colonial Medical Research Committee. Eleventh Annual Report (1955-1956). 1956;312 – pp. |
