# Supplementary material for: A Systematic Review of Mortality from Untreated Scrub Typhus (Orientia tsutsugamushi)
Source: PLoS Negl Trop Dis. 2015 Aug 14;9(8):e0003971. doi: 10.1371/journal.pntd.0003971 (PMC4537241; doi:10.1371/journal.pntd.0003971)
Supplement: S3 Table — (DOCX) [file pntd.0003971.s007.docx]

**Supplementary Table 3: Reasons for article exclusion**

| **Study title** | **Reason for exclusion** | | | | | | **Notes** |
| --- | --- | --- | --- | --- | --- | --- | --- |
|  | **Diagnosis not clear** | **Treated with antibiotics / serum** | **<10 Patients** | **Lacks information on treatment / diagnosis / outcome** | **Repeated patient cohort** | **Asymptomatic** |  |
| Agress CCM. Clinical Survey of Eighty-Six Cases of Scrub Typhus. Bull United States Army Med Dep. 1944;V(2):163–9. | N | N | N | N | Y | N | Cohort repeated in "Sayen JJ, Pond HS, Forrester JS, Wood FC. Scrub Typhus in Assam and Burma. A Clinical Study of 616 Cases. Medicine (Baltimore). 1946;25(2):155–214. " and " Mackie T, Davis G, Fuller H, Knapp J, Steinacker M, Stager K, et al. Observations on Tsutsugamushi Disease (Scrub Typhus) in Assam and Burma. Am J Hyg. 1946;43(3):195–218." |
| Andrew R. A note on the incubation period of scrub types and its correlation with clinical severity. Med J Aust. 1945;2:335. | N | N | N | N | Y | N | Cohort repeated in "Southcott R V. Observations on the epidemiology of tsutsugamashi disease in North Queensland. Medical Journal of Australia (Sydney). 1947. p. 441–50. " |
| Ashburn PM, Craig CF. A comparative study of tsutsugamushi disease and spotted or tick typhus of Montana. Philipp J Sci. 1908;III:1–29. | N | N | N | N | Y | N | Patient cohort repeated in "Kitashima T. Studien uber die Tsutsugamushi-krankheit. Kitasato Arch Exp Med. 1918;11(2):91." |
| Audy, J. & Harrison, J., 1951. A review of Investigations on Mite Typhus in Burma and Malaya, 1945-50. Transactions of the Royal Society of Tropical Medicine and Hygiene, 44(4), pp.371–404. | N | N | Y | Y | N | N | No patient cohort. Paper focuses on vectors and epidemiology. |
| Audy, J., 1949. Practical notes on Scrub Typhus in the field. Journal of the Royal Army Medical Corps, 936, pp.273–288. | N | N | Y | Y | N | N | No patient cohort. Paper focuses on vectors and epidemiology. |
| Audy JR, Al. E. Scrub Typhus Investigations in South East Asia. A Report on Investigations by G.H.Q. [India] Field Typhus Research Team, and the Medical Research Council Field Typhus Team, based on the Scrub Typhus Research Laboratory South East Asia Command, Imphal. Par. Scrub Typhus Investig South East Asia A Rep Investig by GHQ [India] F Typhus Res Team, Med Res Counc F Typhus Team, based Scrub Typhus Res Lab South East Asia Command Imphal Par. 1947; | N | N | N | N | Y | N | Cohort repeated in "Sayers M, Hill I. The occurrence and identification of the typhus group of fevers in South East Asia Command. J R Army Med Corps. 1948;90(1):6–22." |
| Ashburn PM, Craig CF. A comparative study of tsutsugamushi disease and spotted or tick typhus of montana. Philipp J Sci. 1908;III:1–29. | N | N | Y | N | N | N | Cohort repeated in "Kitashima T. Studien uber die Tsutsugamushi-krankheit. Kitasato Arch Exp Med. 1918;11(2):91. " |
| Bablet J. Premiers résultats d’une enquete sur le typhus exanthématique au Tonkin. Bull la Soc Pathol Exot. 1926;19:766. | Y | N | N | Y | N | N | Prison outbreak. No diagnostics. Likely endemic or epidemic typhus. |
| Bablet J. Syndrome rabiforme dans un cas de typhus chez l’Annamite. Bull la Soc Pathol Exot. 1927;21:218. | Y | N | Y | Y | N | N | Only one case. No clear diagnosis. |
| Bailey CA, Ley HL, Diercks FH, Lewthwaite R, Smadel JE. Treatment of Scrub Typhus: Evaluation of Chloramphenicol, Aureomycin, Terramycin and Para-Aminobenzoic Acid. Antibiot Chemother. 1951;1(1):16. | N | Y | N | N | N | N | All treated with antibiotics. |
| Bengtson IA. A serological study of 37 cases of tsutsugamushi disease (scrub typhus) occurring in Burma and the Philippine Islands. Public Health Rep. 1946;61:887–94. | N | N | N | Y | N | N | No information on patient outcome. |
| Berman, S., Irving, G. & Kundin, W., 1973. Epidemiology of the acute fevers of unknown origin in South Vietnam: effect of laboratory support upon clinical diagnosis. American Journal of Tropical Medicine and Hygiene, 22(6), p.I. | N | Y | N | Y | N | N | No Information on patient outcome or treatment |
| Blake FG. The symptomatology of Tsutsugamushi Disease. In: Moulton FR, editor. Rickettsial Disease of Man. American Association for the Advancement of Science; 1948. p. 147–59. | N | N | N | N | Y | N | Significant overlap with other patient series. |
| Blake F, Maxcy K, Sadusk J, Kohls G, Bell E. Studies on Tsutsugamushi disease (scrub typus, mite-borne typhus) In New Guinea and adjacent Islands. Am J Trop Med Hyg. 1945;41(3):243–373. | N | N | N | N | Y | N | Summary of literature. One cohort of 25 patients repeated from "Lipman BL, Casey A V, Byron RA, Evans EC. Scrub Typhus. Results of a Study of the Cases of Two Hundred Patients admitted to and treated at a Station Hospital between Feb. 9, 1943 and Feb. 4, 1944. War Med (Chic 1941). 1944;6(5):304–15. " |
| Breinl A, Priestley H, Fielding JW. On the occurrence and pathology of endemic glandular fever. A specific fever, occurring in the Mossman district of North Queensland. J Trop Med Hyg. 1915;30. | N | N | N | N | N | N | No clear diagnosis of scrub tyhpus. ?Aetiology of Mossman fever. |
| Breinl A. Mossman Fever. Med J Aust. 1914;1(17):391. | N | N | N | N | Y | N | Cohort repeated in "Breinl A, Priestley H, Fielding JW. On the occurrence and pathology of endemic glandular fever. A specific fever, occurring in the Mossman district of North Queensland. J Trop Med Hyg. 1915;30. " |
| Brown, G.W. et al., 1984. Febrile illness in Malaysia - an analysis of 1,629 hospitalized patients. American Journal of Tropical Medicine and Hygiene, 33(2), pp.311–315. | N | Y | N | Y | N | N | No Information on patient outcome or treatment |
| Brown, G., Robinson, D. & Huxsoll, D., 1976. Scrub typhus: a common cause of illness in indigenous populations. Transactions of the Royal Society of Tropical Medicine and Hygiene, 70(5-6), pp.444–448. | N | Y | N | Y | N | N | No information on patient outcomes. No clear treatment information. "The common practice of prescribing antibiotics (notably tetracycline) probably aborts many infections unrecognized." |
| Bush FK. Typhus Fever in the Simla Hills. JRArmy Med Corps. 1936;67:158. | Y | N | Y | N | N | N | 6 Patients. No clear diagnosis of scrub typhus. |
| Cao, M. et al., 2006. Spring scrub typhus, People’s Republic of China [5]. Emerging Infectious Diseases, 12(9), pp.1463–1465. | N | Y | N | Y | N | N | No information on patient outcome or treatment |
| Card, W.I. & Walker, J.M., 1947. Scrub-Typhus Vaccine : Field Trial in South-East Asia. Lancet, 249(6450), pp.481–483. | Y | Y | N | N | N | N | No clear diagnostic criteria (diagnosis on clinical grounds only). Patients received vaccine of unknown efficacy. |
| Carley, J.G. et al., 1955. The Investigation of Fevers in North Queensland by Mouse Inoculation, with particular reference to Scrub Typhus. Australasian Ann. Med., 4(2), pp.91–99. | N | Y | N | Y | N | N | No information on patient outcome or treatment. Unsure if treated |
| Chang, W. et al., 1990. Seroepidemiological survey of Tsutsugamushi disease in Korea, 1989. Journal of the Korean Society for Microbiology, 25(3), pp.227–235. | N | Y | N | Y | N | N | No clinical information or information on patient outcome or treatment. |
| Chaudhry, D. & Goyal, S., 2013. Scrub typhus-resurgence of a forgotten killer. Indian Journal of Anaesthesia, 57(2), pp.135–136. | N | N | Y | Y | N | N | No patient cohort. Review. |
| Chheng, K. et al., 2013. A prospective study of the causes of febrile illness requiring hospitalization in children in Cambodia. PLoS ONE, 8(4), p.e60634. | N | Y | N | Y | N | N | No information on patients' clinical outcome or treatment. |
| Chrispal, A. et al., 2010. Scrub typhus: an unrecognized threat in South India - clinical profile and predictors of mortality. Tropical Doctor, 40(3), pp.129–133. | N | Y | N | Y | N | N | No Information on patient outcome or treatment |
| Chu, H. et al., 2010. Phylogenetic clustering of 4 prevalent virulence genes in Orientia tsutsugamushi isolates from human patients. Journal of Microbiology, 48(1), pp.124–128. | N | N | Y | Y | N | N | Animal studies. No patient information. |
| Coppin H. Sur une fievre epidemique du Tonkin rappelant le typhus exanthematique. Bull la Soc Med chiurgicale l’Indochine. 1921;66–82. | Y | N | N | Y | N | N | Prison outbreak. No eschar or access to laboratory diagnostics. Likely endemic or epidemic typhus. |
| Corbett AJ. Scrub typhus. Bull United States Army Med Dep. 1943;70:34–54. | N | N | N | Y | N | N | Pathological reports only. No patient cohort. |
| Covell G. Studies on typhus in the Simla Hills. Indian J Med Res. 1936;23(3):701–20. | Y | N | N | Y | N | N | Summary of literature. No patient cohort. |
| De Roda AP. Typhus Fever in the Philippines: I, Weil-Felix Reaction of 500 Febrile Cases. J Philipp Islands Med Assoc. 1937;17:147. | N | N | N | Y | N | N | No information on outcome of Scrub typhus patients |
| Deller, J., 1967. An analysis of fevers of unknown origin in american soldiers in vietnam. Annals of Internal Medicine, 66(6), pp.1129–1143. | N | Y | N | N | N | N | 11 cases but at least 7 treated with antibiotics. |
| Dinger JE. Tropical “Scrub” typhus bij witte muizen. Geneeskd Tijdschr voor Ned. 1933;73:329. | N | N | Y | Y | N | N | Paper on the diagnostics of Scrub typhus. |
| Doherty, R., 1956. A clinical study of scrub typhus in North Queensland. Medical Journal of Australia, 43(6), pp.212–220. | N | Y | N | N | N | N | Majority of patients treated. (48/53) |
| Duncan GG. Scrub or mite typhus (tsutsugamushi disease, bush typhus, Japanese river fever, Sumatra mite fever, rural tropical typhus, etc.). Med Clin North Am. 1944;28:1464–70. | N | N | Y | N | N | N | Review of disease. No case series. |
| Elsom, K.A. et al., 1961. Scrub Typhus: a Follow-Up Study. Ann. Intern. Med., 55(5), pp.784–795. | N | N | N | Y | N | N | Patients were convalescent and followed up after the disease. |
| Faa, A. et al., 2003. Scrub Typhus in the Torres Strait Islands Australia. Emerging infectious diseases, 9(4), pp.8–10. | N | Y | N | N | N | N | Majority of Patients treated (6/9). |
| Fairley, N.H., 1945. Medicine in Jungle Warfare. Proceedings of the Royal Society of Medicine, 38(5), pp.195–198. | N | N | Y | Y | N | N | No patient cohort. |
| Farinaud ME, Levy G, Frezieres H. Chloromycetin treatment of scrub typhus in Indochina. Med Trop. 1950;10(3):511. | N | Y | N | N | N | N | 7 of 15 patients treated. |
| Farner DS, Katsampes CP. Tsutsugamushi disease. Nav Med Bull. 1944;800. | N | N | N | Y | N | N | Useful review but no separate patient cohort |
| Fletcher, W. & Lesslar, J.E., 1926. Tropical Typhus in the Federated Malay States with a Compilation on Epidemic Typhus. Malayan Medical Journal, 1(2), p.17–pp. | N | N | N | N | Y | N | Repeated cohort (see Fletcher, W. & Lesslar, J.E., 1925. Tropical Typhus in the Federated Malay States with a Compilation on Epidemic Typhus. Bull. Inst. Med. Res., Federated Malay States, (2), p.88–pp.) |
| Fletcher W. Scrub Typhus. Br Med J. 1932;1140. | N | N | N | Y | N | N | Review. No separate patient cohort. |
| Fletcher W. Typhus-like Fevers of Unknown Aetiology, with special Reference to the Malay States. Proceeding R Soc Med. 1930;1021. | N | N | N | Y | N | N | Review. No separate patient cohort. |
| Fletcher W. Typhus Fevers in Malaya. Trans R Soc Trop Med Hyg. 1935;29(2):111–2. | N | N | N | Y | N | N | Review. One cohort of tropical typhus "220 cases with 12% mortality in 1931". However no distinction between murine and scrub typhus in this cohort. |
| Furth. Latest researches into spotted fever (typhus). Trans 2nd Congr Far East Assoc Trop Med. 1914;262. | Y | N | N | Y | N | N | No clear diagnosis. Likely epidemic form. No mention of eschar. |
| Gottfried, S.P., 1945. A Preliminary Study of Blood Chemistry Findings in Scrub Typhus. American Journal of Clinical Pathology, 15(2), pp.71–76. | N | Y | N | Y | N | N | No information on patient treatment or outcome. |
| Gunther CEM. A survey of endemic typhus in New guinea. Med J Aust. 1940;2:564. | N | N | N | Y | Y | N | Majority of patients repeated in "Gunther CEM, Schroeder AG. Further Observations on Endemic Typhus in New Guinea. Med J Aust. 1939;1(18):688–91. " 105 patients with 20 deaths |
| Gunther CEM. Endemic typhus in New Guinea. Med J Aust. 1935;1:813. | N | N | Y | N | N | N | Case report of 2 patients. |
| Gurung, S., Pradhan, J. & Bhutia, P.Y., 2013. Outbreak of scrub typhus in the North East Himalayan region-Sikkim: an emerging threat. Indian journal of medical microbiology, 31(1), pp.72–74. | N | Y | N | N | N | N | Patients all treated. |
| Hazlett, D., 1970. Scrub Typhus in Vietnam: Experience at the 8th Field Hospital. Military Medicine, January, pp.31–34. | N | Y | N | N | N | N | Patients all treated. |
| Heaslip WG. Investigation of condition known as coastal fever in North Queensland, separation from scrub typhus. Med J Aust. 1940;2(22):555. | Y | N | N | N | N | N | Investigations into coastal fever. No clear diagnosis. |
| Heap BJ. Scrub Typhus in Hong Kong. J Trop Med Hyg. 1991;94:97–101. | N | Y | N | N | N | N | All patients treated with antibiotics. |
| Hone FS. A series of cases closely resembling typhus fever. Med J Aust. 1922;1:1. | Y | N | N | Y | N | N | Patients with no clear diagnosis. Presenting in southern Australia so unlikely to be area where scrub typhus is endemic. |
| Hwang, J. & Lee, C., 2010. Risk factors leading to fatal outcome in scrub typhus. JK Science, 12(2), pp.67–69. | N | N | Y | Y | N | N | Review. No patient cohort. |
| Jang, M. et al., 2014. Differences in the clinical presentation and the frequency of complications between elderly and non-elderly scrub typhus patients. Archives of Gerontology and Geriatrics, 58(2), pp.196–200. | N | Y | N | N | N | N | Patients treated. |
| Jensenius, M. et al., 2009. Multicenter GeoSentinel analysis of rickettsial diseases in international travelers, 1996-2008. Emerging Infectious Diseases, 15(11), pp.1791–1798. | N | Y | N | Y | N | N | No clear information on patient treatment. |
| Kalra SL. Scrub Typhus : Variations in Clinical Symptoms and Strains. Indian Medical Gazette. 1947. p. 516–7. | Y | N | N | Y | N | N | No clear history on patient series. Review. |
| Kasper, M. et al., 2012. Infectious etiologies of acute febrile illness among patients seeking health care in South-Central Cambodia. American Journal of Tropical Medicine and Hygiene, 86(2), pp.246–253. | N | Y | N | Y | N | N | No information on patient outcome or treatment. |
| Kawamura R, Ueda M. Eine neue Thereapie der Dementia paralytica. Dtsch Medizinische Wochenschrift. 1940;19(27):689. | N | N | N | N | Y | N | Repeat article in German of "Kawamura R, Ueda M. On the Treatment of General Paresis with the Pescadores Strain of Tsutsugamushi Virus. Kitasato Arch Exp Med. 1939;16(3):183–96. " |
| Kawamura, R. & Yamahuchi, M., 1921. On the Tsutsugamushi Disease in Formosa, together with a Comparative Study of the same in Northern Japan. Ueber die Tsutsugamushi-Krankheit in Formosa, zugleich eine vergleiehende Studie derselben mit der in Nordjapan. The Kitasato archives of experimental medicine, 4(3), p.169–pp. | Y | N | Y | Y | N | N | No clear patient cohort and outcome of patients. (German) |
| Kawamura R. Studies on tsutsugamushi disease (Japanese Flood Fever). Med Bull Coll Med (Uni Cincinnati). 1926;4(1). | N | N | N | N | Y | N | Repeated Cohort "Hara Y, Abe T. The influence of chemotherapy on the mortality rates of tsutsugamushi disease in Northern Japan, and some other statistical information. Am J Trop Med Hyg. Not Available; 1956;5(2):218–23." |
| Kawamura R, Yamamiya C. On the Tsutsugamushi Disease in the Pescadores. Kitasato Arch Exp Med. 1939;16(1):79 – pp. | N | N | N | N | Y | N | Repeated cohort in "Sasa M. Comparative Epidemiology of Tsutsugamushi Disease in Japan (Studies on Tsutsugamushi, Part 76). Jikken Igaku Zasshi = Japanese J Exp Med. 1954;24(6):335–61. " |
| Kim, D.-M. et al., 2010. Clinical and laboratory findings associated with severe scrub typhus. BMC Infectious Diseases, 10(108), p.108. | N | Y | N | N | N | N | Patients all treated. |
| Kim, I.-H. et al., 2010. Scrub typhus in patients with liver cirrhosis: A preliminary study. Clinical microbiology and infection 16(5), pp.419–424. | N | Y | N | N | N | N | Patients all treated. |
| Kingsbury AN. Annual Report of the Institute for Medical Research, Federated States of Malay. 1929; | N | N | N | N | Y | N | Repeated cohort "Anigstein L. Resarches on tropical typhus -. Kyle Palmer and co., LTD., Kuala Lumpur; 1933. " and "1. Allen G V. Annual Report of the Institute for Medical Research for the Year 1928. Fed Malay States Ann, Rep Med Dept. 1928;27 – pp. " |
| Kingsbury AN. Annual Report of the Insitute for Medical Research, Federeated States of Malay. 1930. | N | N | N | N | Y | N | Repeated cohort "Anigstein L. Resarches on tropical typhus -. Kyle Palmer and co., LTD., Kuala Lumpur; 1933. " |
| Kingsbury AN. Annual report of the Institute for medical research for the year 1931. 1931. | N | N | N | Y | N | N | No information on patient outcome. |
| Kingsbury AN. Annual report of the Institute for medical research for the year 1932. 1932. | N | N | N | Y | N | N | No information on patient outcome. |
| Kingsbury AN. Annual report of the Institute for medical research for the year 1932. 1934. | N | N | N | Y | N | N | No patient cohort. |
| Kingsbury, A.N., 1936. Annual Report of the Institute for Medical Research (F.M.S.) for the Year 1935. Annual Report of the Institute for Medical Research (F.M.S.) for the Year 1935. | N | N | Y | N | N | N | Animal studies. No patient cohort. |
| Kingsbury AN. Annual Report of the Insitute for Medical Research, Federeated States of Malay. 1938; | N | N | N | Y | N | N | No information on mortality in patient cohort. |
| Kirschner L, Kuijer A. Diagnosis and Symptoms of Tropical Typhus. Geneeskd Tijdschr voor Ned. 1932;72(3):153–pp. | Y | N | N | N | N | N | Diagnosed as OX-19 strain therefore not consistent with Scrub typhus |
| Kohls GM, Armbrust CA, Irons EN, Cornelius B. Studies on tsutsugamushi disease (scrub typhus, mite-borne typhus) in new guinea and adjacent islands: Further observations on epidemiology and etiology. Am J Epidemiol. 1945;374–96. | N | N | N | Y | Y | N | No clear information on patient cohorts. At least one cohort repeated in "Irons E, Armstrong H. Scrub typhus in Dutch New Guinea. Ann Intern Med. 1947;26(2):201–20. " |
| Kouwenaar W, Wolff JW. Rickettsia in Sumatra. Proc 6th Pacific Sci Congr, 1939. 1942;5:633. | N | N | N | Y | N | N | Review. No patient cohort. |
| Kumar, K. et al., 2004. Outbreak investigation of Scrub Typhus in Himachal Pradesh (India). Journal of Communicable Diseases, 36(4), pp.277–283. | N | Y | N | Y | N | N | No information on treatment. All admitted to hospital and diagnosed as scrub typhus so likely to have been treated. |
| Kumar, V.V. et al., 2014. Scrub Typhus Is an Under-recognized Cause of Acute Febrile Illness with Acute Kidney Injury in India. PLoS Neglected Tropical Diseases, 8(1), p.6. | N | Y | N | N | N | N | Patients all treated. Unwell patients received dialysis. |
| Lagrange E. A propos d’un cas de pseudo-typhus en Annam. Bull la Soc Pathol Exot. 1923;16(2):105. | N | N | Y | N | N | N | Single case report. |
| Lancaster, H.O., 1953. The mortality in Australia from typhus, typhoid fever and infections of the bowel. Medical Journal of Australia, 1(17), pp.576–579. | N | N | Y | Y | N | N | No clear patient cohort with tropical typhus |
| Lee, C. et al., 2009. Risk factors leading to fatal outcome in scrub typhus patients. American Journal of Tropical Medicine and Hygiene, 81(3), pp.484–488. | N | Y | N | N | N | N | Patients all treated. Unwell patients treated on ICU. |
| Legac, P. & Arquie, E., 1964. Endemic factors in scrub typhus in Indochina. Bulletin de la Societe de pathologie exotique et de ses filiales, 57, pp.277–283. | N | Y | N | Y | N | N | 5708 cases and 158 deaths reported but no information on treatment. |
| Leimena J. Een geval van scrubtyphus (tropical typhus). Geneeskd Tijdschr voor Ned. 1941;81:339. | N | N | Y | N | N | N | Only 1 patient. |
| Leishman, A.W.D. & Kelsall, A.R., 1944. A Year of Military Medicine in India. Lancet, 244(6312), pp.231–235. | Y | N | N | Y | N | N | 43 patients (0 deaths) with "tropical typhus" 13/23 tested positive for Weil-Felix OX19 therefore unlikely to all be Scrub typhus. |
| Levine, H.D., 1945. Cardiac Complications of Tsutsugamushi Fever (Scrub Typhus) : an Investigation of their Persistency. War Medicine, 7(2), pp.76–81. | N | N | N | Y | N | N | 130 convalescent patients therefore no information on mortality from original infection. |
| Lewis, M. et al., 2003. Scrub Typhus Reemergence in the Maldives. Emerging Infectious Diseases, 9(12), pp.1638–1641. | Y | Y | N | Y | N | N | 168 patients and 10 deaths. No clear information on treatment. Only 38 cases laboratory confirmed. |
| Lewthwaite, R. & Savoor, S., 1936. The Typhus group of diseases in Malaya. The British Journal of Experimental Pathology, 17(1), p.parts I–VII. | Y | N | Y | N | N | N | No untreated patient cohort |
| Lewthwaite R, Savoor SR. Tropical Typhus (Rural type) and the Tsutsugamushi disease as encountered in the Federated Malay States. Trans 9th Congr Far East Assoc Trop Med. 1934;1:249–57. | N | N | Y | Y | N | N | Summary of diagnostics. No patient cohort. |
| Lewthwaite R. Annual report of the Institute for Medical Research 1936. 1936; | N | N | N | Y | Y | N | No information on patient outcome. Cohort repeated in "Lewthwaite E, Savoor SB. Rickettsia diseases of Malaya: Identity of tsutsugamushi and rural typhus. Lancet. 1940;5:633. " |
| Lewthwaite R. Annual Report of the Insitute for Medical Research, Federeated States of Malay. 1946. | N | N | N | Y | N | N | No information on patient outcome. |
| Ley H., Smadel JE. Antibiotic therapy of rickettsial diseases. Antibiot Chemother. H.L. Ley Jr., Army Med. Serv., Grad. Sch., Washington, DC, United States; 1954;4(7):792–802. | N | N | N | N | Y | N | Repeated untreated cohort "Smadel J, Woodward T, Ley Jr. H, Lewthwaite R. Chloramphenicol (chloromycetin) in the treatment of tsutsugamushi disease (scrub typhus). J Clin Invest. 1949;28(2-5):1196–215." |
| Li, T., Yang, Z. & Wang, M., 2013. Scrub typhus rapidly increased in Guangzhou, Southern China, 2007-2012. Revista do Instituto de Medicina Tropical de Sao Paulo, 55(4), pp.293–294. | N | Y | N | Y | N | N | No clear information on patient treatment or antibiotics. |
| Liang, X. et al., 2014. Hepatic impairment induced by scrub typhus is associated with new onset of renal dysfunction. Clinical Laboratory, 60(1), pp.63–68. | N | Y | N | Y | N | N | (5 deaths in 143 patients) No information on patient treatment or antibiotics. |
| Lipman BL, Byron RA, Casey A V. Clincal survey of scrub typhus fever. Bull United States Army Med Dep. 1944;72:63. | N | N | N | N | Y | N | Patient cohort repeated in "Lipman BL, Casey A V, Byron RA, Evans EC. Scrub Typhus. Results of a Study of the Cases of Two Hundred Patients admitted to and treated at a Station Hospital between Feb. 9, 1943 and Feb. 4, 1944. War Med (Chic 1941). 1944;6(5):304–15. " |
| Liu, Y.-X. et al., 2009. Clinical characteristics of the autumn-winter type scrub typhus cases in south of Shandong province, northern China. BMC Infectious Diseases, 9. | N | Y | N | N | N | N | 480 patients. All patients, apart from 1, were treated. |
| Lowe J. Ten Cases of Typhus in civillians in Calcutta. Ind Med Gaz. 1946;81:171. | N | N | Y | N | N | N | Only 4/10 cases confirmed by serology as Scrub typhus. |
| Machella T., Forrester J. Mite or scrub tyhpus. A clinical and laboratory study of 64 cases. Am J Med Sci. 1945;ccx:38. | N | N | N | N | Y | N | Cohort repeated in "Sayen JJ, Pond HS, Forrester JS, Wood FC. Scrub Typhus in Assam and Burma. A Clinical Study of 616 Cases. Medicine (Baltimore). 1946;25(2):155–214. " |
| Mackie, T. et al., 1946. Observations on Tsutsugamushi Disease (Scrub Typhus) in Assam and Burma. Am. J. Hyg, 43(3), pp.195–218. | N | N | N | N | Y | N | Repeated cohort " Mackie, T. et al., 1946. Observations on Tsutsugamushi Disease (Scrub Typhus) in Assam and Burma. Am. J. Hyg, 43(3), pp.195–218." |
| Maxcy KF. Scrub Typhus (Tsutsugamushi Disease) in the U.S. Army during World War II. In: Moulton FR, editor. Rickettsial Disease of Man. Washington D.C.: American Association for the Advancement of Science; 1948. p. 36–46. | N | N | N | N | Y | N | Significant overlap with other patient series. |
| Mahajan, R., Singh, N. & Kapoor, V., 2010. Antibiotic use in scrub Typhus: Systematic Review and Meta-analysis of clinical trials. JK Science, 12(2), pp.92–94. | N | Y | N | Y | N | N | Meta-analysis of antibiotic treatment. No clear patient cohort |
| Mahajan, A. & Tandon, V., 2010. Scrub typhus-reemergence in Jammu. JK Science, 12(2), pp.55–56. | N | Y | N | N | N | N | 20 patients. All treated with antibiotics. |
| Maitra GC. A Note on cases of Typhus fever in Burma and their distribution. Ind Med Gaz. 1936;71:572. | N | N | N | Y | N | N | No information on patient outcome |
| May AJ. Endemic typhus in Papua. Med Jour Aust. 1941;1:449. | Y | N | Y | N | N | N | Only 2 cases. |
| McGovern V. Pathological Aspects of Scrub Typhus in New Guinea. Med J Aust. 1945;2(5):146–9. | N | N | N | N | Y | N | Repeated cohort "Sangster CB, Kay HB. Scrub typhus: Clinical aspects. Med J Aust. 1945;2:138." |
| Megaw, J., 1921. A typhus-Like Fever in India, Possibly Transmitted by Ticks. The Indian Medical Gazette, October, pp.361–371. | Y | N | N | Y | N | N | Clinical diagnosis only. No evidence of eschar on patients and no record of mortality. Thought to be transmitted by Ticks. |
| Megaw J, Shettle F, Roy D. Typhus-Like Fever, probably tick-typhus in Central India. Ind Med Gaz. 1925;70:53. | Y | N | N | Y | N | N | Clinical diagnosis only. No evidence of eschar on patients. Thought to be transmitted by Ticks. |
| Megaw JWD, Sundar Rao S. Tick-Typhus and other sporadic fevers of the typhus group. Ind Med Gaz. 1928;63:306–18. | Y | N | Y | Y | N | N | No clear diagnosis. Case series with cohorts containing less than 10 patients. |
| Mueller, T. et al., 2014. Acute undifferentiated febrile illness in rural Cambodia: A 3-year prospective observational study. PLoS ONE, 9(4). | N | Y | N | N | N | N | Patients all treated. |
| Nanda, S., Varma, M. & Vidyasagar, S., 2012. Clinical profile of scrub typhus. International Journal of Infectious Diseases, 16, p.e267. | N | Y | N | N | N | N | 269/300 patients treated. No separate information on untreated cohort. |
| Noad KB. Tsutsugamushi fever in natives. Med J Aust. 1946;2:20. | N | N | Y | N | N | N | Only 3 cases described. |
| Noc F, Gautron. Deux cas de fièvre indéterminé rappelant le pseudo-typhus de Delhi observés à Saigon. Bull la Soc Med Chiurgicale l’Indochine. 1915;6(3). | N | N | Y | N | N | N | Only 2 cases. |
| Ogawa, M. et al., 2002. Scrub typhus in Japan: Epidemiology and clinical features of cases reported in 1998. American Journal of Tropical Medicine and Hygiene, 67(2 SUPPL.), pp.162–165. | N | Y | N | N | N | N | No information on treatment but as clinically diagnosed by local clinicians patients are all very likely to be treated. |
| Palm TA. Some Account of a Disease called “Shima-Mushi” or “Island-Insect Disease”, by the Natives of Japan; peculiar, it is believed, to that country, and hitherto not described. Edinb Med J. 1878;24(1):128–32. | N | N | Y | N | N | N | Only 6 patients included. |
| Philip CB, Kohls GM. Studies on tsutsugamushi disease (scrub typhus, mite-borne typhus) in new guinea and adjacent islands. Am J Epidemiol. 1945;42(2):195–203. | N | N | N | N | Y | N | Cohort repeated in "Kohls GM, Armbrust CA, Irons EN, Cornelius B. Studies on tsutsugamushi disease (scrub typhus, mite-borne typhus) in new guinea and adjacent islands: Further observations on epidemiology and etiology. Am J Epidemiol. 1945;374–96." |
| Philip CB. Observations on tsutsugamusiii disease (mite- borne or scrub typhus) in northwest honshu island. Japan, in the fall of 1945. Am J Hyg. 1947;4:45–59. | N | N | N | N | Y | N | Cohort repeated in "Hara Y, Abe T. The influence of chemotherapy on the mortality rates of tsutsugamushi disease in Northern Japan, and some other statistical information. Am J Trop Med Hyg. Not Available; 1956;5(2):218–23. " |
| Philip CB. Tsutsugamushi disease (scrub typhus) in World War II. J Parasitol. C.B. Philip, Rocky Mountain Lab., Hamilton, MT, United States; 1948;34(3):169–91. | Y | N | N | Y | Y | N | No clear information on diagnostics. Crude analysis of incidence and mortality from scrub typhus. Majority of patients are included in other cohorts. Excluded to prevent duplication. |
| Plooij, M., 1950. Scrub typhus. Nederlands Tijdschrift voor Geneeskunde, 2(21), pp.1490–1498. | N | Y | N | Y | N | N | No information on mortality. (10/40 treated). |
| Prezyna, A.P. et al., 1954. Treatment of Scrub Typhus in the Pesadores Islands with Chlorampheniool, Aureomycin and Terramycin. American Journal of Tropical Medicine and Hygiene, 3(4), pp.608–614. | N | Y | Y | N | N | N | 47/50 treated patients. 3 untreated patients excluded as cohort too small. |
| Punjabi, N.H. et al., 2012. Etiology of acute, non-malaria, febrile illnesses in Jayapura, northeastern Papua, Indonesia. The American journal of tropical medicine and hygiene, 86(1), pp.46–51. | N | Y | Y | N | N | N | No information on precise management but likely to be given antibiotics. Only 7 patients. |
| Ragiot C, Delbove P. Typhus endémique bénin en Cochinchine. Bull la Soc Pathol Exot. 1934;881. | N | N | Y | Y | N | N | No clear diagnosis. ? Murine typhus. |
| Ragiot C, Delbove P. Typhus endémique et typhus tropical en Cochinchine. Bull la Soc Pathol Exot. 1935;31:163. | N | N | Y | N | N | N | Likely scrub typhus but only 3 cases reported. |
| Ragiot C. Note au sujet des typhus typhus dits “tropicaux” observés en Indochine méridonale. Bull la Soc Pathol Exot.1938;31:460. | N | N | N | Y | N | N | Not enough information on cases and outcome |
| Ragiot C, Delbove P. Les fièvres exanthémique du type “tsutsugamushi” en Indochine méridionale. Proc 6th Pacific Sci Congr, 1939. 1942;5:623. | N | N | N | Y | N | N | No patient cohort. |
| Rivoalen, Bruneau & Kernevez, 1939. A Fatal Case of Tropical Typhus contracted in the Haut-Laos District of French Indo-China. (Typhus tropical mortel contracte dans le Haut-Laos.). Revue Medicale Francaise d’Extreme-Orient, (10), pp.1125–1126. | N | N | Y | N | N | N | Single case report. |
| Sadusk, J.F., 1947. Typhus fever in the Untied States Army following immunization. Incidence, severity of the disease, modification of the clinical course and serologic diagnosis. JAMA (Chicago, Ill.), 133(16), pp.1192–1199. | Y | N | N | Y | N | N | Discusses 5 cases with epidemic typhus |
| Schuffner WAP. Pseudo-typhus in Deli. Trans 3rd Congr Far East Assoc Trop Med. 1913;309. | N | N | N | N | Y | N | Repeated cohort " Schuffner WAP. Pseudotyphoid Fever in Deli, Sumatra (A variety of Japanese Kedani Fever). Philipp J Sci. 1915;10:345. " |
| Shapiro, M., Dandurov, Y. & Sobolev, I., 1969. Clinical picture of tsutsugamusi’s fever in the primorie territory. Klinicheskaya Meditsina, 47(5), pp.106–110. | N | Y | N | Y | N | N | No clear treatment information but likely treated. (Russian). |
| Silpapojakul, K.K. et al., 2004. Paediatric scrub typhus in Thailand: A study of 73 confirmed cases. Transactions of the Royal Society of Tropical Medicine and Hygiene, 98(6), pp.354–359. | N | Y | Y | N | N | N | Only 5 untreated patients of 73. |
| Singh, R., 2010. Clinical manifestations & complications of scrub typhus. JK Science, 12(2), pp.76–78. | N | N | Y | Y | N | N | No clear patient cohort. (Review) |
| Singh, S.I. et al., 2010. An outbreak of scrub typhus in Bishnupur district of Manipur, India, 2007. Tropical Doctor, 40(3), pp.169–170. | N | Y | N | Y | N | N | 2 deaths in 38 patients. Only 18 patients treated with antibiotics but no information on whether fatal cases treated or not. |
| Smadel, J. et al., 1949. Chloramphenicol (chloromycetin) in the chemoprophylaxis of scrub typhus (tsutsugamushi disease). II. Results with volunteers exposed in hyperendemic areas of scrub typhus. American Journal of Hygiene, 50(1), pp.75–91. | N | Y | N | N | N | N | Patients all treated with chloramphenicol. |
| Smadel, J.E., Bailey, C.A. & Diercks, F.H., 1950. Chloramphenicol (chloromycetin) in the chemoprophylaxis of scrub typhus (tsutsugamushi disease) (III) Suppression of overt disease by prophylactic regimens of four-week duration. American Journal of Hygiene, 51(5), pp.229–241. | N | Y | N | N | N | N | Patients all treated with chloramphenicol. |
| Smadel, J.E., Bailey, C.A. & Diercks, F.H., 1950. Chloramphenicol (chloromycetin) in the chemoprophylaxis of scrub typhus (tsutsugamushi disease) (IV) Relapses of Scrub Typhus in Treated volunteers and their Prevention. American Journal of Hygiene, 51(5), pp.229–241. | N | Y | N | N | N | N | Patients all treated with chloramphenicol. |
| Smadel, E., 1950. Chloramphenicol (chloromycetin) and tropical medicine. Transactions of the Royal Society of Tropical Medicine and Hygiene, 43(6), pp.555–582. | N | N | N | N | Y | N | Same cohort as "Smadel, J. et al., 1949. Chloramphenicol (chloromycetin) in the treatment of tsutsugamushi disease (scrub typhus). Journal of Clinical Investigation, 28(2-5), pp.1196–1215. " |
| Smadel, J. et al., 1948. Chloromycetin in the treatment of scrub typhus. Science, 108(2798), p.489. | N | N | N | N | Y | N | Same cohort as "Smadel, J. et al., 1949. Chloramphenicol (chloromycetin) in the treatment of tsutsugamushi disease (scrub typhus). Journal of Clinical Investigation, 28(2-5), pp.1196–1215. " |
| Smithson O. Mossman Fever. J Trop Med Hyg. 1910;13(23):351–2. | N | N | N | Y | Y | N | Cohort repeated in "Breinl A, Priestley H, Fielding JW. On the occurrence and pathology of endemic glandular fever. A specific fever, occurring in the Mossman district of North Queensland. J Trop Med Hyg. 1915;30. " |
| Spicer, P., Taufa, T. & Benjamin, A., 2007. Scrub typhus (Orientia tsutsugamushi), spotted fever (Rickettsia australis) and dengue fever as possible causes of mysterious deaths in the Strickland Gorge area of Southern Highlands and West Sepik Provinces of Papua New Guinea. Papua and New Guinea medical journal, 50(3-4), pp.172–183. | Y | N | N | Y | N | N | Serological survey with no clear diagnosis and incidence. |
| Summers DH. An outbreak of scrub typhus among Air Force troops in the Southwest Pacific area. Air Surg Bull. 1945;2:371–3. | N | N | N | N | Y | N | Cohort repeated in "Irons E, Armstrong H. Scrub typhus in Dutch New Guinea. Ann Intern Med. 1947;26(2):201–20." |
| Suputtamongkol, Y. et al., 2009. Epidemiology and clinical aspects of rickettsioses in Thailand. H. K.E. et al., eds. Rickettsiology and Rickettsial Diseases Fifth International Conference, 1166, pp.172–179. | N | Y | N | Y | N | N | 4/7 deaths untreated. No information of proportion of whole cohort who were untreated. |
| Swamy, T. & Dutta, B., 1953. Epidemiology of XK typhus in Jamshedpur. Indian Medical Gazette, 88(10), pp.522–525. | N | Y | N | N | Y | N | No clear information on treatment. Overlap with cohort from " Khan, N., 1950. Scrub typhus (As seen in Jamshedpur). Indian Journal of Medical Sciences, 4(11), pp.487–495." |
| Thap, L.C. et al., 2002. Septic shock secondary to scrub typhus: characteristics and complications. The Southeast Asian journal of tropical medicine and public health, 33(4), pp.780–786. | N | Y | N | N | N | N | All patients treated with antibiotics. |
| Thompson AH. A new endemic area of scrub typhus in Japan. Bull United States Army Med Dep. 1949;9:871. | N | N | N | Y | N | N | No record of patient treatment. |
| Unwin M. “Coastal fever” and endemic tropical typhus in North Queensland: Recent investigation, clinical and laboratory findings. Med J Aust. 1935;2:303. | Y | N | N | Y | N | N | No clear information on number of patients or mortality. |
| Varghese, G.M. et al., 2013. Scrub typhus in South India: Clinical and laboratory manifestations, genetic variability, and outcome. International Journal of Infectious Diseases, 17(11), pp.e981–7. | N | Y | N | Y | N | N | Patients treated or received ICU care |
| Varghese, G.M. et al., 2006. Scrub typhus among hospitalised patients with febrile illness in South India: Magnitude and clinical predictors. Journal of Infection, 52(1), pp.56–60. | N | Y | N | Y | N | N | Majority of patients treated with antibiotics and no clear distinction between treated and untreated cohorts. |
| Varghese, G.M. et al., 2014. Clinical profile and improving mortality trend of scrub typhus in South India. International Journal of Infectious Diseases, 23, pp.39–43. | N | Y | N | N | N | N | Received antibiotics or ICU care. |
| Vaz, L. & Gupta, N., 2006. Outbreak of scrub typhus in Jammu - A report. Medical Journal Armed Forces India, 62(4), pp.342–343. | N | Y | N | N | N | N | Patients all treated with tetracyclines. |
| Vielle E, Souchard. Sur un cas de typhus exanthématique observé en Cochinchine. Bull la Soc Pathol Exot. 1931;24:302. | N | N | Y | N | N | N | Only one case. Confirmed scrub typhus |
| Von der Borch E. Non-epidemic typhus: a report of fourteen cases occurring in the gold fields, Wau, mandated territory of New Guinea between January 1, 1935, and June30, 1936. Med J Aust. 1937;1:435. | N | N | N | N | Y | N | Repeated Cohort "Gunther CEM, Schroeder AG. Further Observations on Endemic Typhus in New Guinea. Med J Aust. 1939;1(18):688–91." |
| Walch EW, Keukenschrijver NCR. On Pseudo-typhus of Sumatra - Part II - Some Notes on Epidemiology. Proc 5th Congr Far East Assoc Trop Med 1932. 1924;1:627. | N | N | N | N | Y | N | Repeated Cohort in "Walch EW, Keukenschrijver NCR. Eenige opmerkingen aangaande de Epidemiologie van de Pseudotyphus. Geneeskd Tijdschr voor Ned. 1924;63:247. " |
| Walker WT. Scrub Typhus Vaccine. Its effect on sixteen cases incubating the disease. Br Med J. 1947;484–7. | N | Y | N | N | N | N | Patients received experimental vaccine before infection. |
| Wang, Y.-C. et al., 2013. Scrub typhus cases in a teaching hospital in Penghu, Taiwan, 2006-2010. Vector-Borne and Zoonotic Diseases, 13(3), pp.154–159. | N | Y | N | N | N | N | Patients all treated. |
| Watt, G. et al., 1996. Scrub typhus infections poorly responsive to antibiotics in northern Thailand. Lancet, 348(9020), pp.86–89. | N | Y | N | N | N | N | Patients all treated. |
| Wei, Y. et al., 2014. Rapid increase of scrub typhus: An epidemiology and spatial-temporal cluster analysis in Guangzhou City, Southern China, 2006-2012. PLoS ONE, 9(7). | N | Y | N | Y | N | N | No information on treatment or patient outcome. |
| Wheatland FT. Wheatland Mossman Fever Med.J.Aus 1924.pdf. Suppl to Med J Aust. 1924;322–5. | N | N | N | N | Y | N | Repeated in "Breinl A, Priestley H, Fielding JW. On the occurrence and pathology of endemic glandular fever. A specific fever, occurring in the Mossman district of North Queensland. J Trop Med Hyg. 1915;30. " and "Derrick E, Berry A, Tonge J, Brown H. Fevers of the Mackay District, Queensland. Med J Aust. 1953;2(4):121–9. " |
| Wisseman, C.L.J. et al., 1954. Studies on Cortisone and Antibiotics for Prompt Therapeutic Control of Typhoid Fever and Scrub Typhus. Journal of Clinical Investigation, 33(2), pp.264–275. | N | Y | N | N | N | N | Patients all treated with antibiotics. |
| Woodhead, L. & Dutta, V., 1941. A note on fever of the typhus group in assam. Indian Medical Gazette, 76, pp.406–410. | N | N | Y | Y | N | N | Only 8 patients and no information on patient outcome. |
| Yamamoto, S. et al., 2000. Annual incidence of tsutsugamushi disease caused by different serotypes of Orientia tsutsugamushi in Miyazaki Prefecture in 1991-1999. Japanese Journal of Infectious Diseases, 53(3), pp.126–127. | N | Y | N | Y | N | N | No information on treatment or patient outcome. |
| Yasunaga, H. et al., 2011. Delay in tetracycline treatment increases the risk of complications in Tsutsugamushi disease: data from the Japanese Diagnosis Procedure Combination database. Internal Medicine, 50(1), pp.37–42. | N | Y | N | N | N | N | All treated although worse prognosis with late treatment. |
| Yi, K.S. et al., 1993. Scrub typhus in Korea: importance of early clinical diagnosis in this newly recognized endemic area. Military medicine, 158(4), pp.269–273. | N | Y | N | N | N | N | All patients treated with Antibiotics. |
| Yersin A, Vassal JJ. Typhus Fever in Indo-China. Philipp J Sci. 1908;131. | Y | N | N | Y | N | N | No clear diagnosis. Epidemiological and clinical features not completely consistent with scrub typhus |
| Yoo, J. et al., 2014. Unusual genotypic distribution of Orientia tsutsugamushi strains causing human infections on Jeju Island. The American journal of tropical medicine and hygiene, 90(3), pp.507–510. | N | Y | N | N | N | N | Patients all treated. |
| Zair AH. Scrub typhus. J R Nav Med Serv. 1944;30:135. | N | N | Y | Y | N | N | Review. No patient cohort. |
