# Supplementary material for: A Systematic Review of Mortality from Untreated Scrub Typhus (Orientia tsutsugamushi)
Source: PLoS Negl Trop Dis. 2015 Aug 14;9(8):e0003971. doi: 10.1371/journal.pntd.0003971 (PMC4537241; doi:10.1371/journal.pntd.0003971)
Supplement: S4 Table — When studies contained more than one patient series, each series was displayed separately. All available data are included, but if data were not available it is indicated by a “-“. Percentage and (number/total number of patients) are quoted, but if no patient number was quoted then a “NA” is used. (DOCX) [file pntd.0003971.s008.docx]

| Supplementary Table 4: Characteristics of included studies, arranged chronologically within each region. When studies contained more than one patient series, each series was displayed separately. All available data are included, but if data were not available it is indicated by a “-“. Percentage and (number/total number of patients) are quoted, but if no patient number was quoted then a “NA” is used. | | | | | | | | | | | | | |
| --- | --- | --- | --- | --- | --- | --- | --- | --- | --- | --- | --- | --- | --- |
| Reference | **County, Year of Study** | | | **Study design** | **Untreated Patients**  **(Number included/total number in series)** | **Median (unless specified) age (range) (number of patients)** | **% Male** | **Median (unless specified)Duration of Fever**  **Days (Range)**  **(Number of patients)** | **Diagnostic test** | **Eschar % (Number)** | **Adeno-pathy % (Number)** | **Complications** | **Crude Mortality**  **% (Number)** |
| India and Burma, Sri Lanka | | | | | | | | | | | | | |
| (MacNamara 1935) Ind. Med. Gaz. | | Simla, India 1932-3 | Retrospective case series. Soldiers | | 16 (16/16) | 22 (6-46) (16/16) | 93.8 (15/16) | 13 (8-21) (16/16) | (15/16) OXK ≥1:150  (1/16) Clinical diagnosis | 0% (0/16) | - | 6.2% (1/16) Pneumonia | 6.3% (1/16) |
| (Boyd 1935) J.R.A.M.C | | India 1934 | Prospective case series. Soldiers | | 20 (20/108)  Murine typhus and MacNamara cases excluded | - | 100% (20/20) | Mean 14.2 (-) (20/43) | (35/43) OXK ≥1:250  (8/43) Clinical diagnosis | - | - | 10% (2/20) Pneumonia  15% (3/20) Delirium | 0% (0/20) |
| (Bardhan 1944)  Ind. Med. Gaz | | Kumaon Hills, India  1937-8 | Retrospective case series  Soldiers | | 11 (11/11) | - | 100% (11/11) | “Majority” 12-14 (-) (11/11) | (11/11) OXK ≥1:125 | 0% (0/11) | - | 9.1% (1/11) Patient pneumonia | 0% (0/11) |
|  |  | Central India  1937-8 | Retrospective case series  Soldiers | | 30 (30/30) | - | 100% (30/30) | - | (30/30) OXK ≥1:250 | 0% (0/30) | - | - | 0% (0/30) |
| (Singh 1945)  Ind. Med. Gaz. | | Burma  Sept 1941 | Retrospective case series  Soldiers | | 107 (107/107) | - | 100% (107/107) | “Majority” 13-18 (-) (107/107) | (107/107) OXK ≥1:100 | 35.6% (38/107) | 82.2% (88/107) | 6% (7/107) Epistaxis | 1.9% (2/107) |
| (Hay 1944)  J.R.N.M.S | | Addu Atoll  October-November 1941 | Retrospective case series  Soldiers | | 50 (50/50) | - | 100% (50/50) | “Majority” 12-20 (-) (NA/50) | (50/50) OXK ≥1:320 | 6% (3/50) | 6% (3/50) | 2% (1/50) Bronchopneumonia | 0% (0/50) |
| (Sayers & Hill 1948)  J.R.A.M.C | | Calcutta, India  Autumn 1942 | Retrospective case series | | 16 (16/16) | - | 100% (16/16) | - | (16/16) OXK “positive” | - | - | - | 25% (4/16) |
|  |  | Mandalay, Burma  Autumn 1943 | Retrospective case series  Japanese Soldiers, | | 80 (80/80) | - | 100% (80/80) | - | (7/44) OXK “significant titre”  (73/80) Clinical diagnosis | - | - | - | 7.4%  (-/80) |
|  |  | Northern Burma  May 1944 | Retrospective case series  British soldiers | | 50 (50/50) | - | 100% (50/50) | - | (50/50) Clinical diagnosis | - | - | - | 30% (15/50) |
| (Lusk 1945)  Ind. Med. Gaz. | | Calcutta, India  Jun – Dec 1943 | Retrospective case series  Military hospital | | 97 (97/114)  (Murine excluded) | - | 100% (97/97) | 57.9% 12-20 (6-35) (60/60) | (96/114) OXK ≥1:125 | 0% (0/97) | 2.1% (2/97) | 1% (1/97) Bronchopneumonia  1% (1/97) Deafness  2.1% (2/97) Delirium | 11.3% (11/97) |
| (Tattersall 1945)  Lancet | | India / Burma  Oct 1943-Jan1944 | Retrospective case series  Indian and European Soldiers | | 1000 | 28 (NA/1000) | 100% (1000/1000) | 18 (-) (NA/1000) | (500/500) OXK ≥1:200  (500/1000) Clinical diagnosis | 11% (56/500) | 92% (460/500) | 10% (50/500) Lobar pneumonia  35% (175/500) Deafness  9% (45/500) Haemorrhagic complication | 6.0% (60/1000) |
| (Sayen et al. 1946)  Medicine | | India and Burma  Oct 1943 – Feb 1945 | Retrospective case series  American and Chinese Soldiers | | 553 (553/616)  Convales-cent patients excluded | -  100% | (553/553) | (10-36) (200/200) | (112/200) OXK ≥1:100  (514/616) Clinical diagnosis | 60% (120/200) | 20% (40/200) | 28% (56/200) Haemorrhage | 5.2% (29/553) |
| (Mackie 1946)  A.J.T.M.H | | India Burma  Nov 1943-Jul 1945 | Retrospective case series  American soldiers | | 694 (694/694) | - | 100% (694/694) | - | No record of method of diagnosis | - | - | - | 8.4% (58/694) |
|  |  |  | Retrospective case series  Chinese soldiers | | 403 (403/403) | - | 100% (403/403) | - | No record of method of diagnosis | - | - | - | 9.9% (40/403) |
| (Willcox 1948)  T.R.S.T.M.H | | Assam + Burma  Jan 1944-Apr 1946 | Retrospective case series  African and British Soldiers | | 493 (493/493) | - | 100% (493/493) | 17.2 (Mean) (400/493) | (63/100) OXK ≥1:125 | 22.0% (99/450) | 90% (NA/450) | 24.5% (98/400) Deafness | 8.3% (41/493) |
|  |  | Ceylon epidemic  Jan 1944 | Retrospective case series  British soldiers | | 756 (756/756) | - | 100% (756/756) | 15 (Mean) (8-30) (756/756) | No record of method of diagnosis | 85%  (NA/450) | - | Headache 77%  Body ache 62% | 1.3% (11/756) |
| (Deshmukh 1945)  E. Afr. Med J. | | 150 General Hospital, Burma  Sept-Dec 1944 | Retrospective case series  East African Soldiers | | 615 (615/615) | - | 100% (615/615) | (7-36) (615/615) | “Most” OXK ≥1:100  “Some” diagnosed clinically | 17.5% (35/200) | 100% (200/200) | 1.5% (3/200) Lobar pneumonia  4% (8/200) Marked cerebral disturbance | 1.9% (12/615) |
| (Louveaux 1947)  Ann. Soc. Bel. Med. Trop. | | Belgian Hospital, Burma  Sept – Dec 1944 | Retrospective case series  East African Soldiers. | | 51 (51/51) | - | 100% (51/51) | 14 (10-30) (51/51) | (21/21) OXK ≥1:25  (30/51) Clinical diagnosis | 2.0% (1/51) | 100% (51/51) | 4% (2/51) Bronchopneumonia | 17.6% (9/51) |
| (Klein 1945)  J. R. Arm. Med. Corp. | | Northern Burma  Oct-Dec 1944 | Retrospective case series  British and Indian Soldiers | | 225 (225/225) | - | 100% (225/225) | 14 (11-25) (225/225) | (225/225) Clinical diagnosis | 34% (14/41) | 100% (41/41) | 2.4% (1/41) Lobar pneumonia  48.8% (20/41) Deafness  12.2% (5/41) mental disturbance | 5.3% (12/225) |
| (Donegan 1945)  Br. J. Opthal. | | Arakan, India/Burma Border  Nov-Dec 1944 | Retrospective case series  West African Soldiers | | 101 (101/101) | 27 (54/101) | 100% (101/101) | 10 (4-27) (98/101) | (97/98) OXK “≥1:50”  (4/101) Clinical diagnosis | - | - | 32% (32/101) “Vitreous haze”  3% (3/101) Pneumonia | 4.0% (4/101) |
| (Menon & Ibbotson 1945)  BMJ | | N.E. Burma  Nov 1944-Jan 1945 | Retrospective case series  British Soldiers | | 110 (110/110) | - | 100% (110/110) | 15.7 (mean) (5-29) (110/110) | (90/110) OXK ≥1:125 (20/110) Clinical diagnosis | 56% (62/110) | 98% (108/110) | 6.4% (7/110) Bronchopneumonia | 8.2% (9/110) |
| (Tierney 1946)  JAMA | | Assam, India  1945-6 | Randomised Control Trial  US soldiers | | 16 (16/34)  (PABA treated patients excluded) | 26 (range 20-37) | 100% (16/16) | 19 (15-79) (16/16) | (33/34) OXK ≥1:80  (1/34) Clinical diagnosis | 75% (12/16) | - | 62.5% (10/16) Pneumonia  31.3% (5/16) Delirium | 18.8% (3/16) |
| (Soman 1954)  J. Ind. Med. Ass | | Mumbai, India  1946-50 | Prospective case series  (1946-50) | | 21 (21/21) | 30 (10-60)(17/21) | 66.7% (14/21) | - | (21/21) OXK ≥1:100  (21/21) *O. tsutsugamushi* isolated in mice | 16.7% (3/18) | 23.1% (3/13) | 14.3% (3/21) Delirium  14.3% (3/21) Bronchopneumonia | 14.3% (3/21) |
| (Reddy 1947)  Ind. Med. Gaz | | North Burma  May-Nov 1946 | Retrospective case series  British Soldiers | | 68 (68/68) | “Majority” 20-30 | 100% (68/68) | “Majority” 18-20 (12-26) (68/68) | (68/68) OXK ≥1:640 | 40% (27/68) | 80% (54/68) | 50% (34/68) Deafness  4% (3/68) Pneumonia | 4.4% (3/68) |
| (Krishnan 1949)  Ind. Med. Gaz | | India, Barrackpore  1946-8 | Retrospective case series  Local Population | | 72 (72/102)  (Murine typhus excluded) | - | - | - (10-22) (72/72) | (46/72) OXK ≥1:125  “36%” Animal Inoculation. | 6.9% (5/72) | 65% (NA/72) | 4.2% (3/72) Delirium  25% (18/72) Deafness | 2.8% (2/72) |
| (Khan 1950)  Ind. J. Med. Sci. | | India, Jamshedpur  1947-9 | Retrospective case series  Local population | | 378 (378/400)  (22 treated patients excluded) | - | 72.1% (270/374) | - (16-42) (378/378) | (378/378) OXK ≥1:250 | “30%”  (NA/378) | “80%”  (NA/378) | “20%” (NA/378) Bronchopneumonia | 2.9% (11/378) |
| (Chaudhuri & Chakravarti 1949)  Ind. Med. Gaz | | India, Calcutta  1947-8 | Retrospective case series  Local Population | | 21 (21/25)  (OX 19 excluded) | 11-61  (15/25) | 61.9% (13/21) | - (14-35) (21/21) | (21/21) (OXK ≥1:100) | 8% (2/25) | 28% (7/25) | 64% (13/21) Deafness  38% (8/21) Bronchopneumonia | 9.5% (2/21) |
| (Singh et al. 2008)  J.M.S. | | India  2006 | Retrospective Outbreak investigation  Local population | | 24 (24/24)  “Most untreated” | - | - | - | (NA/24) OXK ≥1:80  (NA/24) Verbal autopsy of cases. | - | - | ”Severe cases complications of delirium, pneumonitis and myocarditis” | 20.8% (5/24)  50% (2/4) over 45 |
| Japan | | | | | | | | | | | | | |
| (Baelz 1878)  Arch. Pat. Ant. Phys. Clin. Med.  (German) | | Japan  Summer 1877 | Retrospective case series  Local population | | 25 (25/25) | 31 (21-63) (9/25) | - | “2-3 weeks” (-) (25/25) | (25/25) Clinical diagnosis | 100% (25/25) | 100% (25/25) | 8% (2/25) Clinical signs of pneumonia  Delirium “Quite rare” | 12% (3/25) |
| (Kitashima 1918)  Kit. Arch. Exp. Med.  (German) | | Niigata, Japan  1903-1909 | Retrospective case series  Local population | | 1,522 (1,522/1,522) | Median age 21-30 (10month – 82years) (NA/1,522) | 60.0% (780/1299) | “1-3 weeks” (-) (56/1,522) | (1,522/1,522) Clinical diagnosis | 100% (NA/1522) | (56/56) | - | 27.9% (426/1,522) |
| (Tanaka 1906)  Cent. F. Bakt.  (German) | | Akita-Ken, Japan  1905- | Retrospective case series  Local population | | 620 (620/620) | - | - | - | (620/620) Clinical diagnosis | 82.9% (514/620) | 82.9% (514/620) | - | “40%” |
| (Hara & Abe 1956)  AJTMH | | Shinano (Niigata Prefecture) Japan 1917-1948 | Retrospective case series | | 1,267 (1,267/1,267) | - | 61.9% (NA/1,267) | - | (1,267/1,267) Clinical diagnosis | - | - | N.B. Increasing mortality with age seen across all patients. Around 20% in 11-20 increasing to over 50% in those over 50 | 31.6% (400/1267) |
|  |  | Agano (Niigata Prefecture) Japan 1917-1948 | Retrospective case series | | 506 (506/506) | - | 61.9% (NA/506) | - | (506/506) Clinical diagnosis | - | - | - | 45.3% (229/506) |
|  |  | Uono (Niigata Prefecture) Japan 1917-1948 | Retrospective case series | | 220 (220/220) | - | 61.9% (NA/220) | - | (220/220) Clinical diagnosis | - | - | - | 28.6% (63/220) |
|  |  | Mogami (Yamagata Prefecture) Japan 1917-1948 | Retrospective case series | | 83 (83/83) | - | 81.3% (NA/83) | - | (83/83) Clinical diagnosis | - | - | - | 45.8% (38/83) |
|  |  | Omono (Akita Prefecture) Japan 1917-1948 | Retrospective case series | | 698 (698/698) | - | 90.4% (NA/698) | - | (698/698) Clinical diagnosis | - | - | - | 20.2% (141/698) |
| (Berge et al. 1949)  Am J Hyg | | Shinano River, (Niigata Prefecture) Japan 1947-8 | Prospective case series | | 11 (11/20)  vaccinated excluded | - | - | - | (15/20) Isoloation of *O. tsutsugamushi* (5/20) Clinical diagnosis | - | - | - | 18.2% (2/11) |
| (Sasa 1954)  Jin. Ig. Zas. | | Kochi  Sasa & Kawashima (1953) | Summary of case reports  (1919-48) | | 10 (10/10) | - | - | - | (10/10) Clinical diagnosis | - | - | - | 60% (6/10) |
|  |  | Umayado  Kagawa (1955) | Summary of case reports  (1931-49) | | 10 (10/10) | - | - | - | (10/10) Clinical diagnosis | - | - | - | 70% (7/10) |
| Malaysia + Singapore | | | | | | | | | | | | | |
| (Fletcher & Lesslar 1925)  Bul. Ins. Med. Res. Fed. Mal. St. | | Malaysia Aug 1924-Jan 1925 | Retrospective case series | | 18 (18/18) | 25 (11-540) (17/18) | 88.9% (16/18) | 14.5 (10-21) (18/18) | (18/18) ≥1:400 OXK | - | 55.6% (10/18) | 22.2% (4/18) Broncho-pneumonia  44.4% (8/18) Deafness  44.4% (8/18) Pronounced delirium | 0% (0/18) |
| (Fletcher & Lesslar 1926)  J. Trop. Med. Hyg. | | Malaysia 1925-6 | Retrospective case series | | 86 (86/86) | - | - | 14 (most common) – (86/86) | “Always positive”  (86/86) OXK ≥1:100 | “Not present” | “Frequently enlarged” | “35%” Delirium  “Occasional severe pneumonia bronchitis” | 5.8% (5/86) |
| (Fletcher et al. 1929)  Trans. Roy. Soc. Trop. Med. & Hyg. | | Malaysia June 1926- June 1927 | Retrospective case series | | 61 (61/61) | - | - | - | (10/36) “OXK” form of typhus  (19/25) OXK ≥1:500  (32/61) Clinical diagnosis | “0%” (0/61) | “Often enlarged” | - | 9.8% (6/61) |
| (Allen 1928)  An. Rep. Inst. Med. Res. Mal. | | Malaysia  1927-1928 | Retrospective case Series  Rubber Estate Workers | | 84 (84/85)  (Murine typhus cases excluded) | - | - | - | (84/85) OXK (titre unknown)  (3/40) positive inoculation | - | - | - | 7.1% (6/84) |
| (Anigstein 1933)  Kyle, Palmer &Co. | | Malaysia  1929-30 | Prospective case series occurring in Malaysia  June 1929 – Dec 1930 | | 81 (81/147)  (Murine typhus excluded) | - | 91.4% (74/81) | - | (81/81) OXK ≥1:125 | - | - | - | 23.5% (19/81) |
|  |  |  | Prospective case series  Oil palm estate workers | | 90 (90/90) | - | 66.7% (60/90) | Mean 15.7 (10-21) (30/90) | (90/90) OXK ≥1:125 | 0% (0/90) | 55% (49/90) | 16% (14/90) Pneumonic signs  74% (67/90) Deafness | 10.0% (9/90) |
| (Lewthwaite & Savoor 1940)  Lancet | | Malaysia  1930-40 | Retrospective case series  Local population | | 181 (181/250) | - | - | Mean 14-15 days (-) (NA/181) | (179/181) OXK ≥1:125  (2/181) Clinical diagnosis | 7.7% (14/181) | 48.6% (88/181) | 13% (23/181) Bronchopneumonia  4% (7/181) Epistaxis  66% (119/181) Deafness  35% (63/181) Delirium | 7.2% (13/181)  20% (4/20) >40 years old  8.0%(2/25) under 18 |
| (O’Connor 1935)  Mal. Med. Jour. | | Malaysia 1932-4 | Retrospective case series  Local population | | 86 (86/86) | 20-40 (76/86) | 91.2% (79/86) | - | (86/86) “Positive reaction to OXK” (No record of titre) | - | - | 2.3% (2/86) pneumonia | 4.7% (4/86) |
| (Subrahmanyam 1936)  TRSTMH | | Singapore  July 1934 – December 1935 | Prospective case series  Local population | | 16 (16/46)  (Murine cases excluded) | - | 100% (16/16) | - | (16/16) OXK ≥1:125 | - | - | 6.3% (1/16) “Rash” | 6.3% (1/16) |
| (Templeton 1947)  J.R.A.M.C | | Malaysia  Oct 1945 – Feb 1946 | Retrospective case series  British soldiers | | 56 (56/56) | - | 98.2% (55/56) | Mean 15.1 (7-23) (56/56) | “Majority “confirmed through clinical diagnosis. OXK ≥1:80 used to confirm diagnosis in other cases. | 28.6% (16/56) | 100% (56/56) | 12.5% (7/56) Bronchopneumonia  Deafness “not present” | 5.4% (3/56) |
| (Smadel et al. 1949)  J. Clin. Inv. | | Military hospital Kuala Lumpur Malaysia  1948 | NRCT | | 19 (19/58)  Treated patients excluded | 30 (17-55) | 84.2% (16/19) | 14 (12-31) (19/19) | (19/19) OXK ≥1:160  (1/3) Isolation of *O.tsutsugamushi* | 13% (6/47) | - | 10.5% (2/19) Bronchopneumonia  5.3% (1/19) Suppurative parotitis | 5.3% (1/19) |
| New Guinea, New Britain, Soloman Islands | | | | | | | | | | | | | |
| (Gunther 1940)  Med. J. Aus. | | Western Papua New Guinea and New Britain  1930-37 | Summary of case reports  Local population | | 105 (105/105) | - | 90.5% (95/105) | - | (NA/105) confirmed with OXK  (NA/105) Clinical diagnosis | - | - | (2/2) pregnant women aborted | 19.0% (20/105) |
| (Williams et al. 1944)  Med. J. Aus. | | Papua New Guinea  Sep 1942-Sep 1943 | Retrospective case series  Australian Soldiers | | 626 (626/626) | - | 100% (626/626) | 16 (4-40) (626/626) | (500/582) OXK ≥1:125  (126/626) Clinical diagnosis | 59% (375/626) | 66% (409/626) | 14.4% (90/626) Deafness  10.7% (67/626) Pneumonia | 9.7% (61/626)  14% (14/101) in over 30s |
| (Greenfield 1946) Ann. Int. Med. | | Buna Gona campaign Papua New Guinea  1942-3 | Retrospective case series  US soldiers | | 25 (25/25) | - | 96% (24/25) | Mean 11.3 (3-20) (25/25) | (21/25) OXK ≥1:40  (4/25) Clinical diagnosis alone | 80% (20/25) | 80% (20/25) | 4% (1/25) Bronchopneumonia | 0% (0/25) |
| (Irons & Armbrust Jr. 1946)  Bul. US. Army. Med. Dep | | 363 Military Hospital, Port Moresby Papua New Guinea  1944? | Retrospective Case series  US Soldiers | | 74 (74/74) | - | 100% (74/74) | Mean 18 (-) (74/74) | 86% (64/74) OXK ≥1:80. (8/74) Clinical diagnosis | 89% (66/74) | 100% (74/74) | - | 9% (7/74) |
| (Berry et al. 1945)  War Medicine | | Papua New Guinea  Dec 1942 – June 1944 | Retrospective case series  US Soldiers | | 85 (85/195)  Patients admitted for convalescence excluded | - | 100% (85/85) | - (10-31) (85/85) | OXK rising or ≥1:160 “almost invariably positive” | 80% (72/85) | “Major-ity” | 15% (9/60) Major pneumonic signs | 2.4% (2/85) |
| (Ahlm & Lipshutz 1944)  JAMA | | SW Pacific  1943 | Retrospective case Series  US Soldiers | | 70 (70/70) | - | 100% (70/70) | - | (34/70) Clinical  (36/59) OXK ≥1:160 | 67.1%  (47/70) | 94.3%  (66/70) | (47/70) Pneumonic signs  (24/70) Deafness  (14/70) Epistaxis | 1.4% (1/70) |
| (Blake et al. 1945) Am J Pub. Health. | | Dobadura, Papua New Guinea.  Aug-Dec 1943 | Retrospective case series  US soldiers | | 248 (248/248) | - | 100% (248/248) | “Majority 12-14 days” (248/248) | OXK ≥1:160 in the “majority of cases” | - | - | - | 2.5% (6/248) |
| (Lipman et al. 1944)  War Medicine | | Southwest Pacific  Feb 1943-Feb1944 | Retrospective case series | | 200 (200/200) | 25.7 years (mean) (200/200) | 100% (200/200) | “Majority 14-18 days” (NA/200) | (198/200) OXK ≥1:40  (2/200) Clinical diagnosis | 80% (160/200) | 98% (196/200) | 9% (18/200) Bronchopneumonia | 10% (20/200)  (40% in 29 patients over 40) |
| (Sather & Silberstein 1945) Bul. US. Arm. Med. Dept. | | New Guinea  August – November 1943 | Retrospective case series  US soldiers | | 14 (14/14) | - | 100% (14/14) | “Majority 13-15 days” (14/14) | (14/14) OXK >1:160 | 7% (1/14) | 29% (4/14) | 7% (1/14) Bronchopneumonia | 0% (0/14) |
| (Sangster & Kay 1945)  Med. J. Aus. | | New Guinea  Oct.1943 – May 1944 | Retrospective case series  Australian Soldiers | | 235 (235/235) | - | 100% (235/235) | 14-17 days “In majority” | OXK used but no record of proportion | 41% (96/235) | 100% (235/235) | 0.4% (1/235) Bronchopneumonia | 8.5% (20/235) |
| (Ripley 1946)  Arc. Neur. Psyc. Chic | | Good-enough Island New Guinea Nov 1943 – Jan 1944 | Retrospective case series  US Soldiers | | 51 (51/51) | 23-38 years (8/51) | 100% (51/51) | - | (35/39) OXK ≥1:80  (16/51) Clinical diagnosis | 76.4% (39/51) | “Com-mon feature” | 66.7% (34/51) Deafness  56.9% (29/51) Delirium | 25.5% (13/51) |
| (Logue 1944)  Nav. Med. Bul. | | New Britain  Jan1944-Apr1944 | Retrospective case series  US soldiers | | 230 (230/230) | - | 100% (230/230) | - | (230/230) Clinical diagnosis | “Usually found” | “Usually found” | “Death resulted from myocarditis and bronchopneumonia” | 9.6% (22/230) |
| (Anderson & Wing 1945)  War Medicine | | ?Biak Island Papua New Guinea May-June 1944 | Retrospective case series  Melanesian Soldiers | | 49 (49/49) | - | 100% (49/49) | 7-17 days (49/49) | OXK “Usually negative” | 70% (NA/49) | 90% (NA/49) | 15% (NA/49) Deafness | 0% (0/49) |
| (Browning et al. 1945)  AJTMH | | ”Dutch New Guinea” Indonesia  May-Dec 1944 | Retrospective case series  US soldiers | | 173 (173/173) | - | 100% (173/173) | 14-21 days for 80% (3-32) (NA/173) | “Mostly” OXK ≥1:80 (multiple samples) | 80% (NA/173) | 80% or more (NA/173) | 17% (NA/173) Broncho-pneumonia 20% (NA/173) Deafness 1% (NA/173) Encephalitis | 3.5% (6/173) |
| (Irons & Armstrong 1947)  Ann. Int. Med. | | Owi and Biak Islands New Guinea  June-Oct 1944 | Retrospective case series  US Soldiers | | 1255 (1255/1255) | - | 100% (1255/1255) | 13.7 mean (1-30 days) (483/1255) | (1003/1255) OXK (252/1255) Clinical diagnosis | 51% (640/1255) | 97% (NA/1255) | 1.3% (NA/1255) Boncho-pneumonia  0.8% (NA/1255) Deafness  4.1% (NA/1255) Delirium/Convulsions | 0.6 % (8/1255) |
| (Griffiths 1945)  J. Para. | | Vogelkop pennisula New Guinea  July-Sept. 1944 | Retrospective case series  US Soldiers | | 931 (931/931) | - | 100% (931/931) | - (11-21) (275/931) | (931/931) Clinical Diagnosis | “92%” (253/275) | “95%” (261/275) | “1%” (3/275) Delirium | 3.7% (34/931) |
| (Mendell 1946)  Am. J. Med. Sci. | | North New Guinea, Indonesia  Aug-Oct 1944 | Retrospective case series  US Soldiers | | 75 (75/87)  Exclusion as no clear diagnosis | - | 100% (71/71) | 10-20 (61%) (7-34 days) (46/75) | (29/32) OXK ≥1:40  (46/75) Clinical diagnosis | 68% (51/75) | 95% (71/75) | 34.7% (26/75) Deafness  35% (26/71) Deafness | 2.7% (2/75) |
| (De Vidas 1945)  Med. J. Aus. | | Papua New Guinea  Year | Retrospective case series  Australian Soldiers | | 80 (80/80) | - | 100% (80/80) | - | (63/80) “Clinical Diagnosis”  (17/80) OXK rising or ≥1:160 | 100% (80/80) | 100% (80/80) | - | 25% (20/80) |
| Australia | | | | | | | | | | | | | |
| (Derrick et al. 1953)  Med. J. Aus. | | Queensland, Australia  1915-22 | Retrospective historical case series  “Sarina Fever” | | 53 (53/53) | - | - | - | (53/53) Clinical diagnosis. Negative typhoid and malaria investigations | “No eschar “ | “Often enlarged” | Some patients had “fatal termination from heart failure” | 18.9% (10/53) |
| (Mathew 1938)  Med. Jour. Aus. | | North Queensland Australia 1935-37 | Retrospective case series | | 71 (71/77)  (Murine typhus excluded) | 30 (6-76) (71/71) | 84.5% (60/71) | 14 (10-42) (50/71) | (68/71) OXK ≥1:125  (1/16) Innoculation suggestive of *O. tsutsugamushi*  (3/71) Clinical diagnosis | 36.2% (17/47) | 82.1% (32/39) | - | 5.6% (4/71) |
| (Heaslip 1941)  Med. J. Aus. | | Queensland, Australia  1938-39 | Prospective case series  Local population | | 54 (54/54) | 31 (6-52) (51/54) | 100% (54/54) | 14 (8-23) (54/54) | (37/48) OXK ≥1:160  (27/29) Animal Inoculation  (7/54) clinical diagnosis | 35.4% (17/48) | - | Pneumonia “occasional” | 1.9% (1/54) |
| (Southcott 1947)  Med. J. Aus. | | Queensland, Australia  1944 | Retrospective case series  Australian Soldiers | | 70 (70/70) | - | 100% (70/70) | - | (70/70) OXK ≥1:160 | 82.4% (28/34) | - | - | 1.4% (1/70) |
| (McBride et al. 1999)  Med. J. Aus. | | North Queensland Australia 1996 | Retrospective case series | | 17 (17/28)  (Treated patients excluded) | - | 100% (17/17) | - | (5/6) OXK ≥1:256  (12/17) Clinical diagnosis | 5.9% (1/17) | - | 0% (0/17) Deafness  0% (0/17) Pneumonia  0% (0/17) Delirium | 0% (0/17) |
| Indonesia (Excluding New Guinea) | | | | | | | | | | | | | |
| (Schuffner 1915)  Phil. J. Sci. | | Sumatra, Indonesia,  1908 | Retrospective case series  Local population | | 158 (158/158) | - | - | - | (158/158) “Clinical diagnosis” | 39.9%  (63/158) | 39.9%  (63/158) | “Bronchopneumonia in fatal cases”  “No Delirium or deafness recorded” | “3%” (5/158) |
| (Walch & Keukenschrijver 1924)  G. T. v. N. I.  (Dutch) | | Sumatra, Indonesia  Oct.1922 – Aug. 1923 | Retrospective case series | | 111 (111/111) | - | - | - | (111/111) Clinical diagnosis | “Not always present” | - | - | 3.6% (4/111) |
| (Emanuels 1932)  G. T. v. N. I.  (Dutch) | | Sumatra, Indonesia  1931 | Retrospective case series  Soldiers | | 25 (25/25) | - | 100% (25/25) | 13-17 (12/25) | (13/13) OXK ≥1:200 | 53.8% (7/13) | 83.3% (10/12) | 7.7% (1/13) Pneumonia | 0% (0/25) |
| (Van Der Schroeff 1941)  G. T. v. N. I.  (Dutch) | | Sumatra, Indonesia  1939-40 | Retrospective case series | | 77 (77/77) | - | - | 16 (8-41) (77/77) | (75/77) OXK ≥1:200 | 37.7% (29/77) | 97.4% (75/77) | - | 3.9% (3/77) |
| Taiwan (and Pescadores Islands) | | | | | | | | | | | | | |
| (Hatori 1921)  Trans. 4^th^ Cong. F. E. As. Trop. Med. | | Taiwan  1913-17 | Retrospective case series | | 615 (615/615) | - | 81% (349/431) | “Range 10-21+ days” (NA/615) | (615/615) Clinical diagnosis  Negative Blood culture, typhoid agglutination. | 100% (615/615) | “100% (615/615) | 34% (40/118) Bronchitis | 9.6% (59/615) |
| (Sasa 1954)  Jin. Ig. Zas. | | Taiwan  Morishita (1934) | Retrospective case series  1923-32 | | 878 (878/878) | - | - | - | (878/878) Clinical diagnosis | - | - | - | 10.6% (93/878) |
|  |  | Pescadores  Morishita (1939) | Retrospective case series  1931-38 | | 284 (284/284) | - | - | - | (284/284) Clinical diagnosis | - | - | - | 5.7% (16/284) |
| (Morishita 1942)  Proc. 6^th^ Sci. Pac. Cong. Ass. | | Taiwan  1933-8 | Retrospective case series  Local Population | | 166 (166/166) | Median age 26-30 (166/166) | 83.1% (138/166) | - | (166/166) Clinical diagnosis | - | - | (1923-32 cohort excluded as quoted in Sasa 1954)  (Information on mortality by age) | 20.5% (34/166) |
|  |  | Boko Islands  1933-38 | Retrospective case series  Local Population | | 284 (284/284) | Median age 6-10 years (284/284) | 52.6% (132/251) | - | (284/284) Clinical diagnosis | - | - | - | 5.6% (16/284) |
| Korea | | | | | | | | | | | | | |
| (Weir 1915)  Chin. Med. J. | | Korea  1913-4 | Retrospective case series  Local population | | 15 (15/15) | - | - | “About 16 days” (15/15) | (15/15) Clinical diagnosis | - | - | 13.3% (2/15) Deafness  13.3% (2/15) Epistaxis | 0% (0/15) |
| Philippines | | | | | | | | | | | | | |
| (Philip et al. 1946)  AJTMH | | Philippines  Dec 1944- August 1945 | Retrospective case series  US Soldiers | | 222 (222/222) | - | 100% (222/222) | “10-21 days” (222/222) | (139/222) OXK ≥1:80  (25/222) OXK rise in titre  (58/222) Clinical Diagnosis | 40.5% (90/222) | - | - | 4.5% (10/222) |
| Cambodia + Vietnam | | | | | | | | | | | | | |
| (Delbove et al. 1938)  Bul. Soc. Path. Exot (French) | | Cambodia  1937 | Retrospective case series | | 20 (20/20) | - | - | “10-15 days” (20/20) | (16/20) OXK ≥1:100  (4/20) Clinical Diagnosis | 0% (0/20) | 0% (0/20) | “Pulmonary complications quite frequent” | 15% (3/20) |
| (Berman & Kundin 1973)  Ann. Int. Med. | | Vietnam  1967-69 | Retrospective case series  US Soldiers | | 19 (19/87) Treated patients excluded | - | 100% (19/19) | 14 (-) (19/19) | (19/19) 4-fold rise in IFA | “4%” (NA/19) | “78%” (NA/19) | 3% (NA/19) Pneumonia | 0% (0/19) |
| Artificial Inoculation | | | | | | | | | | | | | |
| (Kawamura & Ueda 1939)  Kit. Arc. Exp. Med. | | Japan (artificial experiment)  1935 | Prospective case series  Patients with syphilis infected with scrub typhus | | 20 (20/20) | 37 (17-45) (20/20) | 70% (14/20) | “7-14 days” (20/20) | (20/20) Artificially inoculated with attenuated *Pescadores* strain of *O.tsutsugamushi* | n/a | - | No other recorded complications | 5% (1/20) |
| (Kawamura et al. 1939) Kit. Arc. Exp. Med. | | Japan (artificial experiment)  1937 | Prospective case series  Volunteers artificially infected with scrub typhus | | 31 (31/31) | 25 (11-61) | 64.5% (20/31) | “About 2 weeks” (31/31) | (31/31) Artificially inoculated with attenuated *Pescadores* strain of *O.tsutsugamushi* | n/a | - | No other recorded complications | 0% (0/31) |
| (Kawamura & Ueda 1941) Z. Ges. Neurol. Psychiat. | | Japan (artificial experiment)  1937-40 | Prospective case series  Patients with syphilis infected with scrub typhus | | 100 (100/100) | 35 (17-67) (100/100) | 82% (82/100) | 9 (2-17) (100/100) | (100/100) Artificially inoculated with attenuated *Pescadores* strain of *O.tsutsugamushi* | n/a | - | No other recorded complications | 0% (0/100) |

Ahlm, C.E. & Lipshutz, J., 1944. Tsutsugamushi Fever in the Southwest Pacific Theater. *Journal of the American Medical Association*, 124(16), pp.1095–1100.

Allen, G. V, 1928. Annual Report of the Institute for Medical Research for the Year 1928. *Federated Malay States Ann, Rep. Med. Dept.*, p.27–pp.

Anderson, W.L. & Wing, W.M., 1945. Tsutsugamushi disease (Scrub Typhus). *War Medicine*, 8, p.163.

Anigstein, L., 1933. *Researches on tropical typhus -*, Kyle Palmer and co., Ltd., Kuala Lumpur.

Baelz, E., 1878. Das japanische Fluss - oder Ueberschwemmungsfleber, eine acute Infectionskrankheit. *archiv fur pathologische anatomie und physilogie und fur klinische medicin*, 78, p.373.

Bardhan, P.N., 1944. Typhus in the United Provinces of India. *Indian Medical Gazette*, 79, p.150.

Berge, T.O., Gauld, R.L. & Kitaoka, M., 1949. A field trial of a vaccine prepared from the Volner strain of Rickettsia tsutsugamushi. *American Journal of Hygiene*, 50, pp.337–342.

Berman, S. & Kundin, W., 1973. Scrub Typhus in South Vietnam. *Annals of Internal Medicine*, 76, pp.26–30.

Berry, M.G., Johnson, A.S.J. & Warshauer, S.E., 1945. Tsutsugamushi fever. Clinical observations in one hundred and ninety-five cases. *War Medicine*, 7, p.71.

Blake, F.G. et al., 1945. Tsutsugamushi Disease (Scrub Typhus, Mite-borne Typhus) in New Guinea. *American Journal of Public Health and the Nation’s health*, 35(11), pp.1121–1130.

Boyd, J.S.K., 1935. Fevers of the Typhus Group in India. *Journal of the Royal Army Medical Corps*, 65(5), p.289.

Browning, J.S. et al., 1945. Scrub typhus. *American Journal of Medical Sciences*, xxv, p.481.

Chaudhuri, R.N. & Chakravarti, H., 1949. Typhus in Calcutta. *Indian Medical Gazette*, 84, pp.43–51.

Delbove, P., Canet, J. & Truong-van-Huan, 1938. A Small Epidemic of Tropical Typhus. (Scrub Typhus) in a Group of Plantations in Cambodia. *Bulletin de la Societe de Pathologie Exotique*, 31(6), p.457–pp.

Derrick, E. et al., 1953. Fevers of the Mackay District, Queensland. *Medical Journal of Australia*, 2(4), pp.121–129.

Deshmukh, M.D., 1945. Clinical Picture of Scrub Typhus in East African Troops on the Burma Front. *East African Medical Journal*, 22(11), pp.360–367.

Donegan, E.A., 1945. Ocular Findings in Tropical Typhus. *British Journal of Opthalmology*, 30, p.11.

Emanuels, B.J., 1932. Enkele onderzoekingen over tropical typhus en (of) mijtekoorts in Atjeh. *Geneeskundig Tijdschrift voor Nederlandsche-Indie*, 72, p.196.

Fletcher, W. & Lesslar, J.E., 1926. Tropical Typhus and Brill’s Disease. *Journal of Tropical Medicine and Hygiene*, 29, pp.374–378.

Fletcher, W. & Lesslar, J.E., 1925. Tropical Typhus in the Federated Malay States with a Compilation on Epidemic Typhus. *Bull. Inst. Med. Res., Federated Malay States*, (2), p.88–pp.

Fletcher, W., Lesslar, J.E. & Lewthwaite, R., 1929. The ætiology of the tsutsugamushi disease and tropical typhus in the Federated Malay States. *Transactions of the Royal Society of Tropical Medicine and Hygiene*, 23(1), p.57.

Greenfield, I., 1946. Tsutsugamushi fever: Agglutination reactions and clinical observations in 25 cases. *Annals of Internal Medicine*, 24, p.192.

Griffiths, J.T., 1945. A Scrub Typhus ( Tsutsugamushi ) Outbreak in Dutch New Guinea Author. *Journal of Parasitology*, 31(5), pp.341–350.

Gunther, C.E.M., 1940. A survey of endemic typhus in New guinea. *Medical Journal of Australia*, 2, p.564.

Hara, Y. & Abe, T., 1956. The influence of chemotherapy on the mortality rates of tsutsugamushi disease in Northern Japan, and some other statistical information. *The American journal of tropical medicine and hygiene*, 5(2), pp.218–223.

Hatori, J., 1921. Tsutsugamushi-Disease in Formosa. *Transactions 4th Congress Far Eastern Assoc. Trop. Med.*, 2, p.187–p.

Hay, C.P., 1944. Scrub typhus at Port “X.” *Journal of the Royal Naval Medical Service*, 40(3), p.127.

Heaslip, W.G., 1941. Tsutsugamushi fever in north Queensland, Australia. *Medical Journal of Australia*, 1, p.380.

Irons, E. & Armstrong, H., 1947. Scrub typhus in Dutch New Guinea. *Annals of Internal Medicine*, 26(2), pp.201–220.

Irons, E.N. & Armbrust Jr., C.A., 1946. Relation of the Weil-Felix Reaction to the Clinical Course of Tsutsugamushi Disease. *Bulletin of the United States Army Medical Department*, 5(1), pp.85–94.

Kawamura, R. et al., 1939. On the Prevention of Tsutusgamushi. *Kitasato Archives of Experimental Medicine*, 16(2), p.93.

Kawamura, R. & Ueda, M., 1941. Eine neue Therapie der dementia paralytica. *Zeitschrift fur die Gesamte Neurologie und Psychiatrie*, 174(3), p.410.

Kawamura, R. & Ueda, M., 1939. On the Treatment of General Paresis with the Pescadores Strain of Tsutsugamushi Virus. *Kitasato Archives of Experimental Medicine*, 16(3), pp.183–196.

Khan, N., 1950. Scrub typhus (As seen in Jamshedpur). *Indian Journal of Medical Sciences*, 4(11), pp.487–495.

Kitashima, T., 1918. Studien uber die Tsutsugamushi-krankheit. *Kitasato Archives of Experimental Medicine*, 11(2), p.91.

Klein, H.S., 1945. An Epidemic of Scrub Typhus. *Journal of the Royal Army Medical Corps*, 85(4), pp.187–190.

Krishnan, K.V., 1949. Clinical features and laboratory diagnosis of XK or mite born typhus as observed in 102 cases in the Barrackpore Area. *Indian Medical*, 84, pp.33–9.

Lewthwaite, E. & Savoor, S.B., 1940. Rickettsia diseases of Malaya: Identity of tsutsugamushi and rural typhus. *Lancet*, 5, p.633.

Lipman, B.L. et al., 1944. Scrub Typhus. Results of a Study of the Cases of Two Hundred Patients admitted to and treated at a Station Hospital between Feb. 9, 1943 and Feb. 4, 1944. *War Medicine*, 6(5), pp.304–315.

Logue, J.B., 1944. Scrub Typhus. Report of Epidemic in the Southwest Pacific. *Naval Medical Bulletin*, 43(4), pp.645–649.

Louveaux, J., 1947. Clinical aspects of scrub typhus on the Burma front. *Annales de la Societe Belge de Medecine Tropicale*, 27, p.105.

Lusk, J., 1945. One hyndred and fourteen cases of typhus fever seein in an indian military hospital in Calcutta. *Indian Medical Gazette*, 80, pp.437–445.

Mackie, T.T., 1946. Observations on tsutsugamushi disease (scrub typhus) in Assam and Burma. *Transactions of the Royal Society of Tropical Medicine and Hygiene*, 40(4), pp.15–56.

MacNamara, C.V., 1935. An Epidemic of Typhus (Vector Unknown) in the Simla Hills. *Journal of the Royal Army Medical Corps*, 64, p.174.

Mathew, R.Y., 1938. Endemic Typhus in North Queensland. *Medical Journal of Australia*, 2(10), p.371–pp.

McBride, W. et al., 1999. Scrub typhus in north Queensland. *Medical Journal of Australia*, 170(7), pp.318–320.

Mendell, T.H., 1946. Scrub Typhus Fever (Tsutsugamushi Disease) in New Guinea. Report of 75 Cases. *American Journal of Medical Sciences*, ccxi, p.481.

Menon, M.C. & Ibbotson, C., 1945. Scrub Typhus : a Clinical Study. *British Medical Journal*, pp.112–114.

Morishita, K., 1942. Tsutsugamushi Disease : its Epidemiology in Formosa. *Proceedings 6th Pacific Sci. Congr., 1939*, 5, p.639–pp.

O’Connor, M.P., 1935. Typhus Fever with Special Reference to its Occurrence in Malaya. *Malayan Medical Journal*, 10(3), pp.78–79.

Philip, C.B., Woodward, T.E. & Sullivan, A.R., 1946. Tsutsugamushi Disease (Scrub or Mite-Borne Typhus) In the Philippine Islands during American Re-occupation in 1944-45. *American Journal of Tropical Medicine and Hygiene*, 26, pp.229–242.

Reddy, D.J., 1947. Scrub typhus in North Burma. *Indian Medical Gazette*, 82(6), pp.330–333.

Ripley, H.S., 1946. Neuropsychiatric Observations on Tsutsugamushi Fever (Scrub Typhus). *Arch. Neurol. Psychiat. Chicago*, 56(1), pp.42–54.

Sangster, C.B. & Kay, H.B., 1945. Scrub typhus: Clinical aspects. *Medical Journal of Australia*, 2, p.138.

Sasa, M., 1954. Comparative Epidemiology of Tsutsugamushi Disease in Japan (Studies on Tsutsugamushi, Part 76). *Jikken Igaku Zasshi (Japanese Journal of Experimental Medicine)*, 24(6), pp.335–361.

Sather, R.O. & Silberstein, J.S., 1945. Scrub Tyhpus. *Bulletin of the United States Army Medical Department*, 4(1), p.68.

Sayen, J.J. et al., 1946. Scrub Typhus in Assam and Burma. A Clinical Study of 616 Cases. *Medicine*, 25(2), pp.155–214.

Sayers, M. & Hill, I., 1948. The occurrence and identification of the typhus group of fevers in South East Asia Command. *Journal of the Royal Army Medical Corps*, 90(1), pp.6–22.

Van Der Schroeff, J.P., 1941. An Epidemic of Mite Fever and Tropical Typhus in Atjeh and Dependencies. *Geneeskundig Tijdschrift voor Nederlandsche-Indie*, 81(20), pp.1103–1122.

Schuffner, W.A.P., 1915. Pseudotyphoid Fever in Deli, Sumatra (A variety of Japanese Kedani Fever). *Philippine Journal of Science*, 10, p.345.

Singh, G., 1945. Report on an epidemic of Scrub Typhus (K form) treated at a General Hospital in Burma. *Indian Medical Gazette*, 80, p.199.

Singh, K. et al., 2008. An outbreak of scrub typhus in Bishenpur district, Manipur 2006. *JMS - Journal of Medical Society*, 22(3), pp.139–142.

Smadel, J. et al., 1949. Chloramphenicol (chloromycetin) in the treatment of tsutsugamushi disease (scrub typhus). *Journal of Clinical Investigation*, 28(2-5), pp.1196–1215.

Soman, D., 1954. Tsutsugamushi Disease (scrub typhus) in Bombay city and suburbs. *Journal of the Indian Medical Association*, 23(9), pp.389–394.

Southcott, R., 1947. Observations on the epidemiology of tsutsugamushi disease in north queensland. *Medical Journal of Australia*, 2, p.441.

Subrahmanyam, C., 1936. Tropical Tyhpus in Singapore. *Transactions of the Royal Society of Tropical Medicine and Hygiene*, 30(2), p.263.

Tanaka, K., 1906. Ueber meine japanische Kedani-Krankheit. *centralblatt fur bakteriologie parasitenkunde und infektionskrankheiten*, 42, p.329.

Tattersall, R., 1945. Tsutsugamushi Fever on the India-Burma Border. *Lancet*, 2, pp.392–394.

Templeton, W., 1947. Scrub typhus in Malaya (clinical and outer notes). *Journal of the Royal Army Medical Corps*, 88(4), pp.153–161.

Tierney, N.A., 1946. Effect of Para-Aminobenzoic acid in tsutsugamushi disease. *Journal of the American Medical Association*, 131, p.280.

De Vidas, J., 1945. A Survey of Scrub Typhus (K Typhus) in New Guinea. *Medical Journal of Australia*, 1(25), pp.631–634.

Walch, E.W. & Keukenschrijver, N.C.R., 1924. Eenige opmerkingen aangaande de Epidemiologie van de Pseudotyphus. *Geneeskundig Tijdschrift voor Nederlandsche-Indie*, 63, p.247.

Weir, H.H., 1915. Weir, H. H. A continued fever of Korea. China Med. Jour., 1915.pdf. *China Medical Journal*, 29, p.307.

Willcox, P.H.A., 1948. Mite Typhus Fever in Assam and Burma, 1944-1946. *Transactions of the Royal Society of Tropical Medicine and Hygiene*, 42(2), pp.171–189.

Williams, S.W., Sinclair, A.J.M. & Jackson, A. V, 1944. Mite-borne (Scrub) Typhus in Papua and the Mandated Territory of New Guinea : Report of 626 Cases. *Medical Journal of Australia*, 2(21), pp.525–539.
