# Supplementary material for: A Systematic Review of Mortality from Untreated Scrub Typhus (Orientia tsutsugamushi)
Source: PLoS Negl Trop Dis. 2015 Aug 14;9(8):e0003971. doi: 10.1371/journal.pntd.0003971 (PMC4537241; doi:10.1371/journal.pntd.0003971)
Supplement: S5 Table — Red reports high risk of bias, yellow medium risk and green low risk of bias. (DOCX) [file pntd.0003971.s009.docx]

**Supplementary Table 5: Risk of bias within studies.** Red reports high risk of bias; yellow medium risk and green low risk of bias.

|  | Patient Selection | Diagnostic Test | Missing data  Information | Outcome |
| --- | --- | --- | --- | --- |
| India / Pakistan / Sri Lanka / Myanmar | | | | |
| MacNamara 1935 |  |  |  |  |
| Boyd 1935 |  |  |  |  |
| Bardhan 1944 (1) |  |  |  |  |
| Bardhan 1944 (2) |  |  |  |  |
| Singh 1945 |  |  |  |  |
| Hay 1945 |  |  |  |  |
| Sayers 1948 (1) |  |  |  |  |
| Sayers 1948 (2) |  |  |  |  |
| Sayers 1948 (3) |  |  |  |  |
| Lusk 1945 |  |  |  |  |
| Tattersall 1945 |  |  |  |  |
| Sayen 1946 |  |  |  |  |
| Mackie 1946 (1) |  |  |  |  |
| Mackie 1946 (2) |  |  |  |  |
| Wilcox 1948 (1) |  |  |  |  |
| Wilcox 1948 (2) |  |  |  |  |
| Deshmukh 1945 |  |  |  |  |
| Louveaux 1947 |  |  |  |  |
| Klein 1945 |  |  |  |  |
| Donegan 1946 |  |  |  |  |
| Menon 1945 |  |  |  |  |
| Tierney 1946 |  |  |  |  |
| Soman 1954 |  |  |  |  |
| Reddy 1947 |  |  |  |  |
| Krishnan 1949 |  |  |  |  |
| Khan 1950 |  |  |  |  |
| Chaudhuri 1949 |  |  |  |  |
| Singh 2008 |  |  |  |  |
| Japan | | | | |
| Baelz 1878 |  |  |  |  |
| Kitashima 1918 |  |  |  |  |
| Tanaka 1906 |  |  |  |  |
| Hara 1956 (1) |  |  |  |  |
| Hara 1956 (2) |  |  |  |  |
| Hara 1956 (3) |  |  |  |  |
| Hara 1956 (4) |  |  |  |  |
| Hara 1956 (5) |  |  |  |  |
| Berge 1949 |  |  |  |  |
| Sasa 1954 (1) |  |  |  |  |
| Sasa 1954 (2) |  |  |  |  |
| Malaysia and Singapore | | | | |
| Fletcher 1925 |  |  |  |  |
| Fletcher 1926 |  |  |  |  |
| Fletcher 1928 |  |  |  |  |
| Allen 1928 |  |  |  |  |
| Anigstein 1933 (1) |  |  |  |  |
| Anigstein 1933 (2) |  |  |  |  |
| Lewthwaite 1940 |  |  |  |  |
| O'Connor 1935 |  |  |  |  |
| Subrahmanyam 1936 |  |  |  |  |
| Templeton 1947 |  |  |  |  |
| Smadel 1949 |  |  |  |  |
| New Guinea | | | | |
| Gunther 1940 |  |  |  |  |
| Williams 1944 |  |  |  |  |
| Greenfield 1946 |  |  |  |  |
| Irons 1946 |  |  |  |  |
| Berry 1945 |  |  |  |  |
| Ahlm 1944 |  |  |  |  |
| Blake 1945 |  |  |  |  |
| Lipman 1944 |  |  |  |  |
| Sather 1945 |  |  |  |  |
| Sangster 1945 |  |  |  |  |
| Ripley 1946 |  |  |  |  |
| Logue 1944 |  |  |  |  |
| Anderson 1945 |  |  |  |  |
| Browning 1945 |  |  |  |  |
| Irons 1947 |  |  |  |  |
| Griffiths 1945 |  |  |  |  |
| Mendell 1946 |  |  |  |  |
| De Vidas 1945 |  |  |  |  |
| Australia | | | | |
| Derrick 1953 |  |  |  |  |
| Mathew 1938 |  |  |  |  |
| Heaslip 1941 |  |  |  |  |
| Southcott 1947 |  |  |  |  |
| McBride 1999 |  |  |  |  |
| Indonesia (Excluding New Guinea) | | | | |
| Schüffner 1915 |  |  |  |  |
| Walch 1924 |  |  |  |  |
| Emanuels 1932 |  |  |  |  |
| Van der Schroeff 1941 |  |  |  |  |
| Taiwan (and Pescadores Islands | | | | |
| Hatori 1921 |  |  |  |  |
| Sasa 1954 (1) |  |  |  |  |
| Sasa 1954 (2) |  |  |  |  |
| Morishita 1939 (1) |  |  |  |  |
| Morishita 1939 (2) |  |  |  |  |
| Korea | | | | |
| Weir 1915 |  |  |  |  |
| Philippines | | | | |
| Philip 1946 |  |  |  |  |
| Cambodia and Vietnam | | | | |
| Delbove 1938 |  |  |  |  |
| Berman 1973 |  |  |  |  |
| Artificial Inoculation | | | | |
| Kawamura 1939 |  |  |  |  |
| Kawamura 1939 |  |  |  |  |
| Kawamura 1941 |  |  |  |  |
