# Supplementary material for: A Systematic Review of Mortality from Untreated Scrub Typhus (Orientia tsutsugamushi)
Source: PLoS Negl Trop Dis. 2015 Aug 14;9(8):e0003971. doi: 10.1371/journal.pntd.0003971 (PMC4537241; doi:10.1371/journal.pntd.0003971)
Supplement: S6 Table — (DOCX) [file pntd.0003971.s010.docx]

**Supplementary Table 6: Further information on bias within studies**

| **Study** | **Patient selection** | **Diagnostic Test** | **Missing Information** | **Outcome** |
| --- | --- | --- | --- | --- |
|  | **Consecutive / Inappropriate exclusions / Difficult to Diagnose patients Excluded?** | **How reliable is the Diagnostic test? (Grade)** | **Blinding / Loss to Follow up / Lacks Information / Selective Outcome Reporting / Unreliable information** |  |
| MacNamara 1935 | Retrospective cases series of 16 patients. No patients excluded from series. | (15/16) OXK ≥1:150 and (1/16) Clinical diagnosis. (Grade III) | No Loss to follow up. Age and sex recorded. No information on adenopathy. | Outcome recorded for all patients |
| Boyd 1935 | Prospective cases series of 20 patients. Patients included in MacNamara series and those with Murine typhus excluded from series. Appropriate exclusions. | (35/43) OXK ≥1:250 and (8/43) clinical diagnosis (Grade III) | No record of sex, adenopathy or eschar. No loss to follow up. | Outcome recorded for all patients |
| Bardhan 1944 (1) | Retrospective case series of 11 patients. No inappropriate exclusions. | (11/11) OXK ≥1:125 (Grade II) | No record of age or adenopathy status, duration of fever or complications. | Outcome recorded for all patients |
| Bardhan 1944 (2) | Retrospective case series of 30 patients. No inappropriate exclusions. | (30/30) OXK ≥1:250 (Grade II) | No record of age or adenopathy status. | Outcome recorded for all patients |
| Singh 1945 | Retrospective case series of 107 patients. No inappropriate exclusions. | (107/107) OXK ≥1:100 (Grade II) | No record of age of patients | Outcome recorded for all patients |
| Hay 1944 | Retrospective case series of 50 patients. No inappropriate exclusions. | (50/50) OXK ≥1:320 (Grade II) | No record of age or accurate information on the duration of fever | Outcome recorded for all patients |
| Sayers 1948 (1) | Retrospective case series of 16 patients. No records of inclusion or exclusion criteria. | (16/16) Positive OXK. No record of titre (Grade III) | No record of age, or any clinical symptoms | Outcome recorded for all patients as a percentage. No exact numbers. |
| Sayers 1948 (2) | Retrospective case series of 80 patients. No records of inclusion or exclusion criteria. | (7/44) "Significant" OXK titre. (73/80) Clinical diagnosis (Grade III) | No record of age, or any clinical symptoms | Outcome recorded for all patients as a percentage. No exact numbers. |
| Sayers 1948 (3) | Retrospective case series of 50 patients. No records of inclusion or exclusion criteria. | (50/50) Patients clinical diagnosis (Grade III) | No record of age, or any clinical symptoms | Outcome recorded for all patients as a percentage. No exact numbers. |
| Lusk 1945 | Retrospective case series of 97 patients. Patients with murine typhus exlcluded. No inappropriate exclusions. | (96/97) OXK ≥1:125 and (1/97) clinical diagnosis (Grade III) | No record of age of patients. | Outcome recorded for all patients |
| Tattersall 1945 | Retrospective case series of 1000 patients. No inappropriate exclusions. | (500/1000) OXK >1:200. (500/1000) Clinical diagnosis. (Grade III) | Detailed record of symptoms for 500/1000 patients. No record for other 500 patients. | Outcome recorded for all patients |
| Sayen 1946 | Retrospective case series of (553/616) consecutive patients. Convalescent patients excluded. | (112/200) OXK >1:100 (514/616) clinical diagnosis. (Grade III) | Detailed information for 200/616 patients. No information for other patients. No record of age of patients. | Outcome recorded for all patients |
| Mackie 1946 (1) | Retrospective case series of 694 patients. No inappropriate exclusions. | No record of diagnostic method (Grade III) | No clinical information | Outcome recorded for all patients |
| Mackie 1946 (2) | Retrospective case series of 403 patients. No inappropriate exclusions. | No record of diagnostic method (Grade III) | No clinical information | Outcome recorded for all patients |
| Wilcox 1948 (1) | Retrospective case series of 493 patients. No inappropriate exclusions. | (63/493) OXK >1:125 (430/493) clinical diagnosis (Grade III) | No information on sex of patient. | Outcome recorded for all patients |
| Wilcox 1948 (2) | Retrospective case series of 756 patients. No inappropriate exclusions. | No information on diagnosis (Grade III) | No clinical information | Mortality recorded as a percentage but no exact figures. |
| Deshmukh 1945 | Retrospective case series of 615 patients. No inappropriate exclusions. | ` "Most" diagnosed through OXK ≥1:100. Others diagnosed clinically. (Grade III) | Clinical information on 200/615 patients. No information on age of patients. | Outcome recorded for all patients |
| Louveaux 1947 | Retrospective case series of 51 patients. No inappropriate exclusions. | (21/21) OXK ≥1:25. (30/51) Clinical diagnosis. (Grade III) | No information on age | Outcome recorded for all patients |
| Klein 1945 | Retrospective case series of 225 patients. No inappropriate exclusions. | (225/225) Clinical diagnosis. (Grade III) | No record of age of patients | Outcome recorded for all patients |
| Donegan 1946 | Retrospective case series of 101 patients. No inappropriate exclusions. | (97/101) OXK>1:50 (4/101) Clinical diagnosis. (Grade III) | No record of eschar or adenopathy | Outcome recorded for all patients |
| Menon 1945 | Retrospective case series of 110 patients. No inappropriate exclusions. | 90/110 OXK ≥1:125 20/110 Clinical diagnosis. (Grade III) | No record of age of patients | Outcome recorded for all patients |
| Tierney 1946 | Prospective, Randomised control trial with 16 untreated patients. Eighteen treated patients excluded. No inappropriate exclusions. Treated patients excluded. | (33/34) OXK ≥1:80 (1/34) Clinical diagnosis (Grade III) | No record of adenopathy | Outcome recorded for all patients |
| Soman 1954 | Prospective case series of 21 patients. Consecutive case series. Only culture positive results included. | 21/21 inoculation of guinea pig (Grade I) | No record of duration of fever | Outcome recorded for all patients |
| Reddy 1947 | Retrospective case series of 68 patients. No inappropriate exclusions | (68/68) OXK ≥1:640 (Grade II) | Comprehensive clinical information | Outcome recorded for all patients |
| Krishnan 1949 | Retrospective case series of 72 patients. Murine typhus excluded. No inappropriate exclusions. | 64/72 OXK >1:125 "36% positive animal inoculation" (Grade II) | No record of age or sex of patients | Outcome recorded for all patients |
| Khan 1950 | Retrospective case series of 378 patients. Twenty-two treated patients excluded. No inappropriate exclusions. | 378/378 OXK >1:250 (Grade II) | No record of age of patients | Outcome recorded for all patients |
| Chandhuri 1949 | Retrospective case series of 21 patients. Four patients with urine typhus excluded. No inappropriate exclusions. | 21/21 OXK >1:100 (Grade II) | Comprehensive clinical information | Outcome recorded for all patients |
| Singh 2008 | Outbreak investigation | "Verbal autopsy" or some patients diagnosed with OXK. (Grade III) | Limited clinical information | Outcome recorded for all patients |
| Baelz 1878 | Retrospective case series of 25 patients. No inappropriate exclusions. | 25/25 clinical diagnosis (Grade III) | No information on sex. Comprehensive clinical information. | Outcome recorded for all patients |
| Kitashima 1918 | Retrospective case series of 1,522 patients. No inappropriate exclusions. | 1522/1522 Clinical diagnosis. (Grade III) | No information on complications | Outcome recorded for all patients |
| Tanaka 1906 | Retrospective case series of 620 patients. No inappropriate exclusions. No clear record of selection criteria. | 620/620 Clinical diagnosis (Grade III) | No information on age / sex / complications. | Mortality recorded as a percentage but no exact figures. |
| Hara 1956 | Retrospective case series of patients reported by doctors. Likely selection bias. No inappropriate exclusions. | (1,267/1,267) Clinical diagnosis (Grade III) | No information on age / eschar / adenopathy or complications | Outcome recorded for all patients |
| Hara 1956 | Retrospective case series of Patients reported by doctors. Likely selection bias. No inappropriate exclusions. | 506 (506/506) Clinical diagnosis (Grade III | No information on age / eschar / adenopathy or complications | Outcome recorded for all patients |
| Hara 1956 | Retrospective case series of Patients reported by doctors. Likely selection bias. No inappropriate exclusions. | 220 (220/220) Clinical diagnosis (Grade III | No information on age / eschar / adenopathy or complications | Outcome recorded for all patients |
| Hara 1956 | Retrospective case series of Patients reported by doctors. Likely selection bias. No inappropriate exclusions. | 83 (83/83) Clinical diagnosis (Grade III | No information on age / eschar / adenopathy or complications | Outcome recorded for all patients |
| Hara 1956 | Retrospective case series of Patients reported by doctors. Likely selection bias. No inappropriate exclusions. | 698 (698/698) Clinical diagnosis (Grade III | No information on age / eschar / adenopathy or complications | Outcome recorded for all patients |
| Berge 1949 | Prospective case series of patients in a scrub typhus vaccine trial. Eleven patients in control group included and 9 in vaccine group excluded. | (15/20) Isolation of O. Tsutsugamushi. (5/20) Clinical diagnosis (Grade III) | No information on age / eschar / adenopathy or complications | Outcome recorded for all patients |
| Sasa 1954 | Summary of case reports. High likelihood of selection bias. | 10/10 Clinical diagnosis. (Grade III) | No demographic or clinical information | Outcome recorded for all patients |
| Sasa 1954 | Summary of case reports. High likelihood of selection bias. | 10/10 Clinical diagnosis. (Grade III) | No demographic or clinical information | Outcome recorded for all patients |
| Fletcher 1925 | Retrospective case series of 18 patients. No inappropriate exclusions. | 18/18 OXK >1:250 (Grade II) | No information on presence of eschar | Outcome recorded for all patients |
| Fletcher 1926 | Retrospective case series of 86 patients. No inappropriate exclusions. | 86/86 OXK positive ≥1:100 (Grade II) | No information on age or sex | Outcome recorded for all patients |
| Fletcher 1929 | Retrospective case series of 61 patients. No inappropriate exclusions | 29/61 OKK positive. 32/61 Clinical diagnosis (Grade III) | No information on age, sex or complications | Outcome recorded for all patients |
| Allen 1928 | Retrospective case series of 84 cases. One murine typus patient excluded. | 84/84 OXK positive. (3/40) Positive inoculation. (Grade III) | No information on demographics or clinical information | Outcome recorded for all patients |
| Anigstein 1933 (1) | Retrospective case series of 81 patients. No inappropriate exclusions. Murine typhus excluded | 81/81 OXK >1:125 (Grade II) | No information on age or clinical information | Outcome recorded for all patients |
| Anigstein 1933 (2) | Retrospective case series of 90 patients. No inappropriate exclusions. | 90/90 OXK >1:125 (Grade II) | No information on age or eschar status | Outcome recorded for all patients |
| Lewthwaite 1940 | 181/250 patients. Sixty Nine patients excluded due to lack of information. | 179/181 OXK >1:125 and 2/181 Clinical diagnosis. (Grade III) | No information on age or sex | Outcome not recorded for all patients due to missing information. |
| O'Connor 1935 | Retrospective case series of 86 patients. No inappropriate exclusions. | 86/86 OXK positive (No record of titre) (Grade III) | No clinical information | Outcome recorded for all patients |
| Subrahmanyam 1936 | Prospective case series 16/46. Thirty patients with murine cases excluded. | 16/16 OXK positive ≥1:125 (Grade II) | No information on age / eschar / adenopathy | Outcome recorded for all patients |
| Templeton 1947 | Retrospective case series of 56 patients. No inappropriate exclusions. | Majority of diagnoses on clinical findings of an eschar. Uncertain cases confirmed by OXK. (Grade III) | No record of age. | Outcome recorded for all patients |
| Smadel 1949 | Controlled trial. Most unwell patients excluded as antibiotics given to those unwell. Patients untreated "when drug not available or if in the late stage of disease when not then desperately unwell". Selection bias. | 19/19 OXK positive ≥1:160 (Grade II) | No record of adenopathy | Outcome recorded for all patients |
| Gunther 1940 | Summary of case reports. Likely selection bias and under reporting of all cases. | Majority clinical diagnosis. Some patients OXK positive. (Grade III) | No information on age / eschar / adenopathy | Outcome recorded for all patients |
| Williams 1944 | Retrospective case series of 626 patients. No inappropriate exclusions. | (500/582) OXK positive ≥1:125 (136/636) Clinical diagnosis (Grade III) | No information on age | Outcome recorded for all patients |
| Greenfield 1946 | Retrospective case series of 25 patients. No inappropriate exclusions. | (21/25) OXK ≥1:40. (4/25) Clinical diagnosis alone. (Grade III) | No information on age | Outcome recorded for all patients |
| Irons 1946 | Retrospective case series of 74 patients. No inappropriate exclusions. | 86% (64/74) OXK ≥1:80. (8/74) Clinical diagnosis. (Grade III) | No information on age | Outcome recorded for all patients |
| Berry 1945 | Retrospective case series of 85 patients. One hundred and ten convalescent patients excluded. | OXK >1:160" almost invariably". (Grade III) | Comprehensive clinical information. No information on age. | Outcome recorded for all patients |
| Ahlm 1944 | Retrospective case series of 70 patients. No inappropriate exclusions. | (36/70) OXK (34/70) Clinical diagnosis. (Grade III) | No information on age or duration of fever | Outcome recorded for all patients |
| Blake 1945 | Retrospective case series of 248 cases. No patients excluded. | OXK >1:160 in the "majority" of cases. (Grade III) | No information on age, eschar, adenopathy or complications | Outcome recorded for all patients |
| Lipman 1944 | Retrospective case series of 200 patients. No inappropriate exclusions | (198/200) OXK >1:40 (2/200) Clinical diagnosis | Comprehensive clinical information for all patients. | Outcome recorded for all patients |
| Sather 1945 | Retrospective case series of 14 patients. No inappropriate exclusions. | (14/14) OXK >1:160 (Grade II) | No information on age | Outcome recorded for all patients |
| Sangster 1945 | Retrospective case series of 235 patients. No inappropriate exclusions | No clear record of proportion diagnosed with OXK. (Grade III) | No record of patient age or complications | Outcome recorded for all patients |
| Ripley 1946 | Retrospective case series of 51 patients. No inappropriate exclusions | (35/39) OXK >1:80 16/51 Clinical diagnosis (Grade III) | No record of duration of fever | Outcome recorded for all patients |
| Logue 1944 | Retrospective case series of 230 patients. No inappropriate exclusions. | (230/230) clinical diagnosis (Grade III) | No record of age or duration of fever | Outcome recorded for all patients |
| Anderson 1945 | Retrospective case series of 49 patients. No inappropriate exclusions. | (49/49) clinical diagnosis. OXK "Usually negative". (Grade III) | No record of age of patients | Outcome recorded for all patients |
| Browning 1945 | Retrospective case series of 173 patients. No inappropriate exclusions. | "Mostly" OXK positive. Remainder clinical diagnosis. (Grade III) | No record of age of patients. | Outcome recorded for all patients |
| Irons 1947 | Retrospective case series of 1,255 patients. No inappropriate exclusions. | (1003/1255) OXK positive (252/1255) Clinical diagnosis. (Grade III) | No record of age of patients | Outcome recorded for all patients |
| Griffiths 1945 | Retrospective case series of 931 patients. No inappropriate exclusions. | (931/931) Clinical diagnosis. (Grade III) | No record of age or duration of fever | Outcome recorded for all patients |
| Mendell 1946 | Retrospective case series of 75 patients. Twelve patients excluded as did not reach the inclusion criteria. No inappropriate exclusions. | (29/75) OXK (46/75) Clinical diagnosis. (Grade III) | No record of age of patients. | Outcome recorded for all patients |
| De Vidas 1945 | Retrospective case series of 80 patients. No inappropriate exclusions but no record of inclusion criteria. | (17/80) OXK (63/80) Clinical diagnosis. (Grade III) | No record of age of patients | Outcome recorded for all patients |
| Derrick 1953 | Historical record of case serie. Likely to have a high degree of bias. | (53/53) Clinical diagnosis. (Grade III) | No record of age, sex or duration of fever. Clinical information not accurate | Outcome recorded for all patients |
| Mathew 1938 | Retrospective case series of 71 patients. Six patients with murine typhus excluded. No inappropriate exclusions. | (68/71) OXK (3/71) Clinical diagnosis. (Grade III) | No record of complications | Outcome recorded for all patients |
| Heaslip 1941 | Prospective investigation of causes of fever with 54 cases. No record of inappropriate exclusions | (7/54) clinical diagnosis (37/54) OXK (27/54) animal inoculation. (Grade III) | No record of adenopathy | Outcome recorded for all patients |
| Southcott 1947 | Retrospective case series of 70 patients. No inappropriate exclusions. | (70/70) OXK ≥1:160 (Grade II) | No information on age, adenopathy or complications | Outcome recorded for all patients |
| McBride 1999 | Retrospective case series of 17 patients. No inappropriate exclusions. | (5/17 OXK ≥1:256) (12/17) Clinical diagnosis. (Grade III) | No information on age or adenopathy | Outcome recorded for all patients |
| Schuffner 1915 | Retrospective case series of 158 patients. No inappropriate exclusions | (158/158) Clinical diagnosis (Grade III) | No information on age, sex or duration of fever | Outcome recorded for all patients |
| Walch 1924 | Retrospective case series of 111 patients. No inappropriate exclusions. | (111/111) Clinical diagnosis (Grade III) | No information on age, adenopathy or complications | Outcome recorded for all patients |
| Emanuels 1932 | Retrospective case series of 25 patients. No inappropriate exclusions. | (25/25) Clinical diagnosis (Grade III) | No information on age of patients | Outcome recorded for all patients |
| Van der Schroeff 1941 | Retrospective case series of 77 patients. No inappropriate exclusions. | (75/77) OXK (2/77) Clinical diagnosis. (Grade III) | No information on age, sex or complications. | Outcome recorded for all patients |
| Hatori 1921 | Retrospective case series of 615 patients. No inappropriate exclusions. | (615/615) Clinical diagnosis(Grade III) | No information on age | Outcome recorded for all patients |
| Sasa 1954 (1) | Retrospective case series. No record of selection Criteria. | (878/878) Clinical diagnosis (Grade III) | No clinical or demographic information | Outcome recorded for all patients |
| Sasa 1954 (2) | Retrospective case series. No record of selection Criteria. | (284/284) Clinical diagnosis (Grade III) | No clinical or demographic information | Outcome recorded for all patients |
| Morishita 1939 (1) | Retrospective case series of 166 patients. No inappropriate exclusions. | (166/166) Clinical diagnosis (Grade III) | Limited clinical information on eschar, adenopathy or complications | Outcome recorded for all patients |
| Morishita 1939 (2) | Retrospective case series of 284 patients. No inappropriate exclusions. | (284/284) Clinical diagnosis (Grade III) | Limited clinical information on eschar, adenopathy or complications | Outcome recorded for all patients |
| Weir 1915 | Retrospective case series of 15 patients. No inappropriate exclusions. | (15/15) Clinical diagnosis (Grade III) | No information on age, sex, eschar or adenopathy | Outcome recorded for all patients |
| Philip 1946 | Retrospective case series of 222 patients. No inappropriate exclusions. | (164/222) OXK positive (58/222 Clinical diagnosis) (Grade III) | No information on age, adenopathy, or complications | Outcome recorded for all patients |
| Delbove 1938 | Retrospective case series of 20 patients. No inappropriate exclusions. | (16/20) OXK positive (4/20) Clinical diagnosis. (Grade III) | No information on age or sex | Outcome recorded for all patients |
| Berman 1973 | (19/87) untreated patients. Treated patients excluded. Most unwell patients treated - therefore selection bias for relatively well patients in this cohort. | (19/19) 4-fold rise in IFA (Grade I) | No information on age | Outcome recorded for all patients |
| Kawamura 1939 | Prospective trial of 20 patients with syphilis. No inappropriate exclusions. | Patients inoculated with disease (Grade I) | No record of adenopathy or complications. | Outcome recorded for all patients |
| Kawamura 1939 | Prospective trial of 31 volunteers innoculated with *O. Tsutsugamushi* | Patients inoculated with disease (Grade I) | No record of adenopathy or complications. | Outcome recorded for all patients |
| Kawamura 1941 | Prospective trial of 100 patients with syphilis. No inappropriate exclusions. | Patients inoculated with disease (Grade I) | No record of adenopathy or complications. | Outcome recorded for all patients |
